# A facile route to ‘naked’ Ag^+^ ions enabling the coordination of the weak Lewis base Ni(CO)_4_

**DOI:** 10.1039/d5sc05589j

**Published:** 2025-11-07

**Authors:** Willi R. Berg, Amina L. Moshtaha, Robin Sievers, Marc Reimann, Tim-Niclas Streit, Susanne M. Rupf, Martin Kaupp, Moritz Malischewski

**Affiliations:** a Institute of Chemistry and Biochemistry, Inorganic Chemistry, Freie Universität Berlin Fabeckstr. 34–36 14195 Berlin Germany moritz.malischewski@fu-berlin.de; b Institut für Chemie, Theoretische Chemie/Quantenchemie Sekr. C7, Technische Universität Berlin Straße des 17. Juni 135 10623 Berlin Germany; c Universität Innsbruck, Institut für Ionenphysik und Angewandte Physik Technikerstr. 25/3 6020 Innsbruck Austria

## Abstract

The reaction of AgCN with two equivalents of tris(pentafluorophenyl)borane (BCF) in CH_2_Cl_2_ or *ortho*-difluorobenzene (*o*DFB) provides a straightforward access to (solvated) Ag^+^ salts of the weakly coordinating cyanide-bridged anion [µ-(CN)(BCF)_2_]^−^ (suggested abbreviation [BCNB]). Reacting Ag[BCNB] with Ni(CO)_4_ yields the first structurally characterized complex in which Ni(CO)_4_ acts as a ligand towards a transition metal ion proving the extremely low basicity of the anion.

## Abstract 2

We report an easy method to generate highly electrophilic Ag^+^ ions in combination with a very weakly-coordinating counteranion. This allowed the first structural characterization of a metal complex featuring Ni(CO)_4_ as a Lewis base.

## Graphical abstract

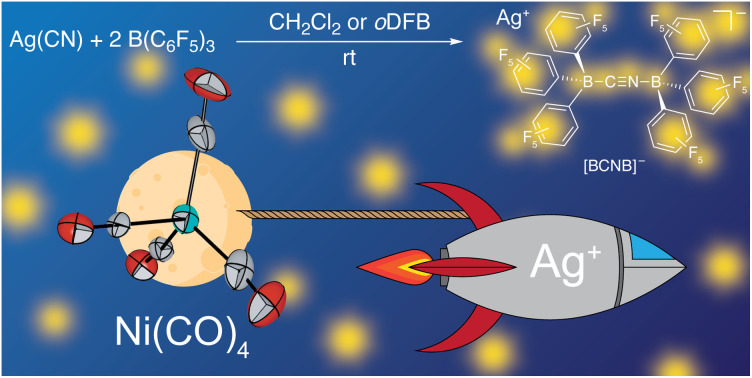

## Introduction

**Fig. 1:**
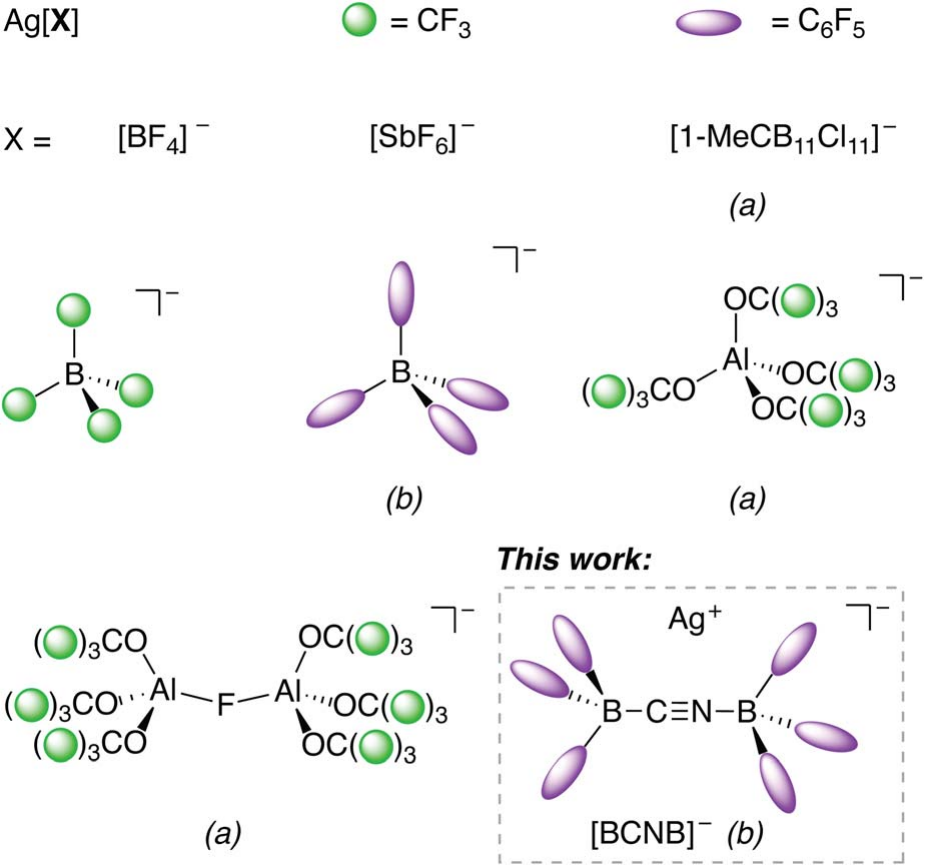
Selected examples of Ag[WCA] salts. (a) Multi-step synthesis procedures required. (b) Not stable as solvent free salt.

Chemically robust, weakly coordinating anions (WCAs) are essential to stabilize reactive cations in both the solution phase as well as in the solid state.^1,2^ When selecting an appropriate WCA, key factors to consider are their oxidative stability,^3^ electrophilic stability^4^ and low coordination ability.^5^ Often these properties are achieved by delocalization of the negative charge over a large non-nucleophilic and chemically robust moiety.^2^ Common WCAs such as borate anions [BF_4_]^−^,^6^ and hexafluorometalates [MF_6_]^−^ (M = As, Sb; and their respective oligomeric structures) are well established.^7,8^ However, they can act as ligands towards highly electrophilic metal centers.^9^ In contrast, “designer” WCAs with improved properties are typically not commercially available and require multi-step syntheses *e.g.* teflate-based anions^10^ or poly-/perfluoro alkoxymetalates.^11–13^ In recent years, [Al(OC(CF_3_)_3_)_4_]^−^ (Fig. 1) showed its effectiveness in stabilizing highly reactive cations.^14^ A multi-step synthesis is required to access its silver salt by salt metathesis of the corresponding Li^+^ salt in liquid SO_2_ (boiling point −10 °C).^11,12^ However, employing such anions enabled the isolation of complexes where two neutral homoleptic transition metal carbonyls such as Fe(CO)_5_ act as ligands towards coinage metals Cu^+^/Ag^+^/Au^+^.^15–18^ Anions such as [SbF_6_]^−^ or [B{3,5-(CF_3_)_2_C_6_H_3_}_4_]^−^ also stabilize the corresponding mono-substituted Fe(CO)_5_ coinage metal adducts,^19–21^ as well as [Hg{Fe(CO)_5_}_2_]^2+^.^22^ Besides mononuclear Fe(CO)_5_ and M(CO)_6_ (M = W, Mo, Nb, Ta)^23,24^ clustered zerovalent homoleptic transition metal carbonyls (Re_2_(CO)_10_, M_3_(CO)_12_ with M = Ru, Os and Ir_4_(CO)_12_) were successfully coordinated to Ag^+^ as well.^25–27^

As indicated by the significantly lower proton affinity of Ni(CO)_4_ (718 kJ mol^−1^, B3LYP-D3(BJ)/def2-TZVPP) in comparison to Fe(CO)_5_ (815 kJ mol^−1^),^8^ use of Ni(CO)_4_ has so far not allowed the structural characterization of a corresponding metal complex in which it acts as a Lewis base although IR spectroscopy hinted to a potential coordination of Ni(CO)_4_ to Ag^+^[FAl(OC(CF_3_)_3_)]^−^ but the potential adduct could neither be isolated in pure form nor crystallized.^28^ To achieve this, a readily available silver salt with a very weakly coordinating counter anion, which can be synthesized in weakly basic solvents, is necessary. In this context, anions derived from fluorinated organoboron Lewis acids are underexplored with respect to their stabilization of sensitive cations in the condensed phase. In principle, anions like [B(CF_3_)_4_]^−^ (Fig. 1), [B(CF_3_)_3_CN]^−^ or [µ-(CN){B(CF_3_)_3_}_2_]^−^ have attractive properties but the B-CF_3_ moiety is only accessible *via* strong fluorinating agents.^29–31^ In contrast, adducts of commercially available B(C_6_F_5_)_3_ (BCF) are far more accessible due to the facile reactions of BCF with nucleophilic anions.^32–38^ Bochmann introduced the cyanide-bridged BCF anion [µ-(CN)(BCF)_2_]^−^ as the respective [CPh_3_]^+^ salt and demonstrated its use in cationic metallocene polymerization reactions.^39,40^ However, the chemistry of this excellent weakly-coordinating anion (for which we propose the abbreviation [BCNB]) has hardly been explored. Interestingly, Ag[BCNB] was never prepared although it could be of great synthetic use for halide abstraction or as an oxidizing agent.^41^ It is conceivable that it may have superior properties in comparison to the structurally related Ag^+^[B(C_6_F_5_)_4_]^−^ (Fig. 1), which has been prepared and is stable only in coordinating solvents (*e.g.* diethyl ether or toluene), as it decomposes in more weakly coordinating solvents (*e.g.* CH_2_Cl_2_), or even as solvent-free salt, to Ag(C_6_F_5_) and B(C_6_F_5_)_3_, limiting its synthetic use.^42^ Ag^+^[B{C_6_H_3_(CF_3_)_2_}_4_]^−^ is prepared in donor solvents such as MeCN, Et_2_O and THF by salt metathesis, multiple filtration steps and requires careful drying *in vacuo*.^43^ The presence of coordinated donor solvents at Ag^+^ had already been hypothesized in the literature and was recently confirmed.^1,44^ In 2025, an easy synthesis for [Ag(C_6_H_6_)_3_]^+^ [B{C_6_H_3_(CF_3_)_2_}_4_]^−^ was reported but this salt slowly decomposes in *o*DFB and rapidly in CH_2_Cl_2_.^44^

## Results and discussion

**Scheme 1:**
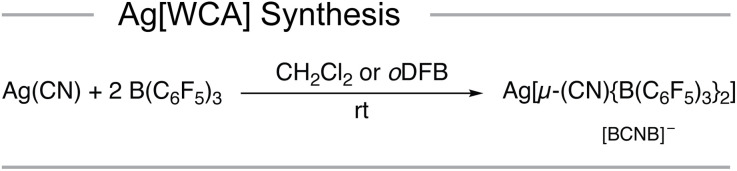
Synthesis of Ag[BCNB] from AgCN with BCF in either CH_2_Cl_2_ or *o*DFB at room temperature.

Here we report on the silver borate salt Ag[µ-(CN)(BCF)_2_] (Ag[BCNB]) which is readily accessible by reacting AgCN with two equivalents of BCF in either CH_2_Cl_2_ (DCM) or *ortho*-difluorobenzene (*o*DFB) at room temperature (Scheme 1).

Both reagents are commercially available and the reaction (which takes place over several hours) is easily followed by dissolution of insoluble AgCN to give a clear colourless solution with almost quantitative conversion. The main advantage of this synthetic route is the *in situ* preparation using only weakly-coordinating solvents without any filtration step. Drying in vacuum gives almost colourless solids which still contain CH_2_Cl_2_ or *o*DFB molecules coordinated to the Ag^+^ center. These silver salts have the advantage over the structurally related Ag[B(C_6_F_5_)_4_] that they can be stored over a long period at room temperature (under inert conditions and exclusion of light).

**Fig. 2:**
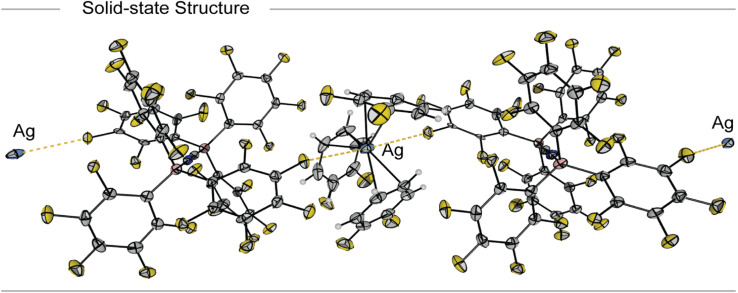
Molecular structure in the solid state of [Ag(*o*DFB)_3_][BCNB]. Displacement ellipsoids are shown at the probability of 50%. Disorder within coordinated *o*DFB molecules and BCF moieties is omitted for clarity. Color code: light-blue = silver, dark-blue = nitrogen, grey = carbon, pink = boron, yellow = fluorine, white = hydrogen.

The ^11^B (Fig. S3 and S12) and ^19^F NMR spectra (Fig. S4 and S11) of the product Ag[BCNB] reveal two different BCF moieties for either the N- or C-bound BCF unit in [BCNB] (^11^B: *δ* = −13 (br, N–B), −23 (br, C–B) ppm, in accordance to the literature).^39^ The CN stretching frequency is observed at *ṽ* = 2290 cm^−1^ in the IR spectrum (Fig. S10). Single crystals of [Ag(*o*DFB)_3_][BCNB] (Fig. 2) were obtained upon layering the solution with *n*-pentane and slowly cooling a saturated solution to −24 °C. It crystallizes in the triclinic space group *P*1̄ and shows that the silver cation is only weakly coordinated by the three *η*^2^-bound *o*DFB solvent molecules and anion [BCNB] with distances in the range of d(Ag–C(*o*DFB)) = 238.2–263.9(3) pm. Each silver center has two Ag–F anion contacts (the closest anion–cation contact is d(Ag–F(BCF)) = 297.6(6) pm), resulting in a chain-like structure (Fig. 2), unlike comparable [Ag(*o*DFB)_2_][Al(OR^F^)_4_] (R^F^ = C(CF_3_)_3_) salts without C–F⋯Ag contacts.^13^

**Fig. 3:**
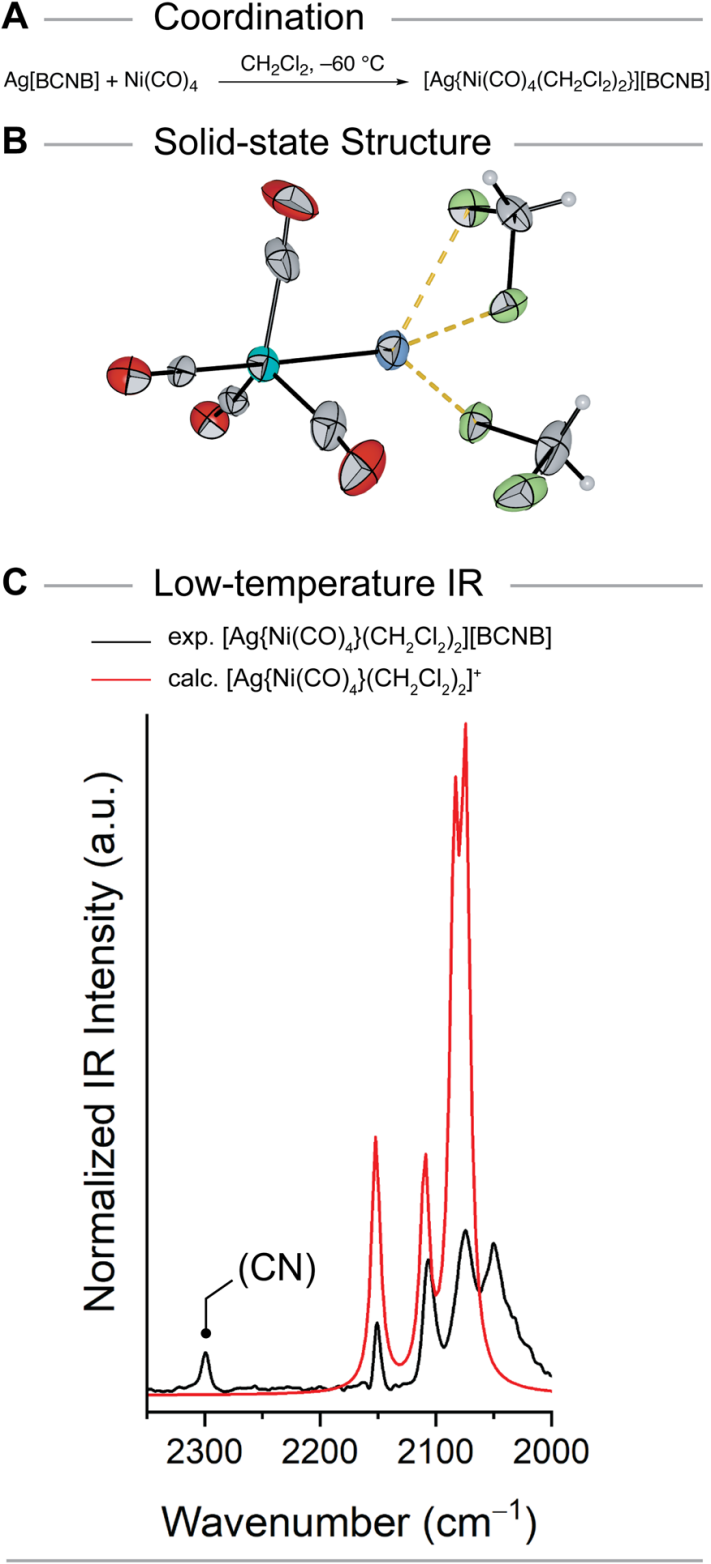
(A) Synthesis of [Ag{Ni(CO)_4_(CH_2_Cl_2_)_2_}][BCNB]. (B) Molecular structure in the solid state of [Ag{Ni(CO)_4_(CH_2_Cl_2_)_2_}]^+^. Displacement ellipsoids are shown at the probability of 50%. Color code: blue = silver, turquoise = nickel, grey = carbon, red = oxygen, green = chlorine, white = hydrogen. Selected bond lengths [pm]: Ag–Ni 258.6(6). (C) Experimental low-temperature IR spectrum of [Ag{Ni(CO)_4_(CH_2_Cl_2_)_2_}][BCNB] (black) compared to the calculated (B3LYP-D3(BJ)/def2-TZVPP) spectrum of [Ag{Ni(CO)_4_(CH_2_Cl_2_)_2_}]^+^ (red) in the region of 2000–2350 cm^−1^. Calculated frequencies are scaled by 0.968 according to Duncan *et al.*.^56^

Although *o*DFB is ideal for the synthesis of electrophilic cations, its relatively high melting point of −34 °C makes the isolation of thermally unstable compounds challenging. For such purposes, the *in situ* generation of Ag[BCNB] in dichloromethane (melting point of −97 °C) is ideal. The synthesis proceeds analogously to the reaction in *o*DFB within hours at room temperature to give a clear solution with almost quantitative conversion. Crystals of [Ag(CH_2_Cl_2_)_3_][BCNB] could be obtained upon cooling to −75 °C but the quality was too poor for publication. However, this source of weakly solvated Ag^+^ proved superior to *o*DFB solutions, as all coordination attempts of Ni(CO)_4_ had to be conducted at −60 °C or below to avoid total decomposition of the metal complexes. Finally, single crystals of the unprecedented [Ni(CO)_4_] → Ag^+^ structural motif could be obtained (Fig. 3A). Layering a DCM solution of Ag[BCNB] and Ni(CO)_4_ with *n*-pentane at −60 °C and slowly cooling the solution to −70 °C gave crystals suitable for single crystal X-ray diffraction. The crystal was identified predominantly containing [Ag{Ni(CO)_4_}(CH_2_Cl_2_)_2_][BCNB] (*ca.* 90%, Fig. S18) with a minor fraction (*ca.* 10%) of [Ag(CH_2_Cl_2_)_3_][BCNB] (Fig. S19). The latter likely arises from partial substitution of Ni(CO)_4_ by the large excess of dichloromethane during the crystallization reflecting the high lability of the system even at low temperature. DFT calculations (Table 1) reveal that this exchange is almost thermoneutral. In the crystal, environmental effects might contribute to the relative stability of [Ag{Ni(CO)_4_}(CH_2_Cl_2_)_2_][BCNB]. In the molecular structure in solid state the counter anion [BCNB] shows substitutional disorder of the {CN} moiety while the fragments {Ag{Ni(CO)_4_}(CH_2_Cl_2_)_2_} and {Ag(CH_2_Cl_2_)_3_} are disordered on two different positions within the asymmetric unit. Despite the intrinsic instability of [Ag{Ni(CO)_4_}(CH_2_Cl_2_)_2_][BCNB], the X-ray data unambiguously confirm the unprecedented Ag–Ni interaction. The cationic unit [Ag{Ni(CO)_4_}(CH_2_Cl_2_)_2_]^+^ (Fig. 3B) shows an Ag–Ni distance of 258.6(6) pm., which is in agreement with the calculated value of 264 pm at the B3LYP-D3(BJ)/def2-TZVPP level. To our knowledge, this represents one of the shortest Ag–Ni bond lengths ever to be reported, even below the values in polynuclear metal clusters.^45–47^ Upon coordination, Ni(CO)_4_ is slightly distorted from its usually tetrahedral structure to a pseudo-trigonal bipyramid. Two of the equatorial CO ligands are slightly bent towards the Ag^+^ center resulting in C_eq._–Ni–Ag angles of 74.2(3), 74.4(2)° and intramolecular Ag–C distances of 272.3(9) and 274.9(6) pm, while the third equatorial CO ligand is slightly more distant at 296.2(5) pm (C_eq._–Ni–Ag angle of 81.9(1)°). Similar interactions have been observed in other metal-only Lewis pairs with Ag^+^.^15,27^ The pseudo trigonal-bipyramidal structure is slightly distorted resulting in a C_ax._–Ni–Ag angle of 169.3(4)°. The C–O distances average to 111.0(4) pm and the Ni–C bond lengths range from 181.4(9) to 185.6(5) pm. Besides the Ni(CO)_4_ moiety, two DCM molecules coordinate to the Ag^+^ center. Furthermore, one additional Ag–F contact to one fluorine atom of the counterion with a distance of 297.9(3) pm is observed. One of the DCM molecules acts as a chelating ligand with two Ag–Cl contacts of 287.0(2) and 289.3(2) pm. The other DCM molecule forms one short (255.7(2) pm) and one very long Ag–Cl contact of 349.0(2) pm which is in the region of the sum of van der Waals radii of Ag and Cl (349 pm).^48^ A quantum chemically optimized structure in the gas phase (at B3LYP-D3(BJ)/def2-TZVPP level) showed a more symmetrical coordination of the two DCM moieties, underlining the importance of the additional Ag–F and Ag–Cl contacts mentioned above for the experimentally observed structure. In general, a variety of silver–dichloromethane complexes have been reported,^49,50^ with Ag–Cl bond lengths varying in the range between 252.4 pm and 302.4 pm.^51,52^ Interestingly, mono- and bidentate coordination modes are frequently observed. For example from solutions of Ag[Al(OC(CF_3_)_3_)_4_] in dichloromethane solvated Ag^+^ cations with 3 chelating or 4 monodentate DCM molecules have been structurally characterized.^51–53^

**Table 1:** Calculated (B3LYP-D3(BJ)/def2TZVPP) reaction enthalpies and Gibbs free energies of Ni(CO)_4_ and Fe(CO)_5_ against Ag^+^ at 298.15 K in the gas phase in kJ mol^−1^

Reaction	L = Ni(CO)_4_			L = Fe(CO)_5_		
	Δ*E*	Δ*H*	Δ*G*	Δ*E*	Δ*H*	Δ*G*
Ag^+^ + L → [Ag-L]^+^	−168.9	−168.6	−140.3	−221.0	−221.3	−188.2
[Ag(CH_2_Cl_2_)_2_]^+^ + L → [(CH_2_Cl_2_)_2_Ag-L]^+^	−79.2	−75.7	−29.1	−109.7	−106.6	−53.8
[Ag(CH_2_Cl_2_)_3_]^+^ + L → [(CH_2_Cl_2_)_2_Ag-L]^+^ + CH_2_Cl_2_	−8.4	−8.7	1.6	−38.9	−39.5	−23.1

[Ag{Ni(CO)_4_(CH_2_Cl_2_)_2_}][BCNB] readily decomposes at temperatures >–40 °C and when exposed to reduced pressure. Raman spectra of crystalline [Ag{Ni(CO)_4_(CH_2_Cl_2_)_2_}][BCNB] were not obtainable due to fluorescence issues. However, it was possible to obtain a low-temperature IR spectrum (Fig. 3C, experimental set up see Fig. S1 and S2), which agrees well with the calculated (B3LYP-D3(BJ)/def2-TZVPP) spectrum, especially considering the neglect of the crystal environment. Both calculated and experimental spectrum reveal four CO stretching frequencies (Table 2 and Fig. 3C, S17), which nicely fit to one another. The CO frequencies within [Ag{Ni(CO)_4_(CH_2_Cl_2_)_2_}]^+^ are blue shifted compared to non-coordinated Ni(CO)_4_ (IR: *ṽ* [cm^−1^] = 2057).^54^ Nevertheless, the complex classifies as a classical carbonyl complex since the average value for *ṽ*(C–O) (exp. 2095/calc. 2105 cm^−1^) is below 2143 cm^−1^.^55^ Attempts to synthesize a moiety in which Ag^+^ is solely coordinated by Ni(CO)_4_ (mono- or bi-coordinated) employing less coordinating solvents (*o*DFB, 1,2,3,4-tetrafluorobenzene and CH_2_ClF) were unsuccessful so far.

**Table 2:** Comparison of experimental and calculated CO stretching frequencies in the region of 2000–2300 cm^−1^. IR spectrum of [Ag{Ni(CO)_4_(CH_2_Cl_2_)_2_}][BCNB] obtained at circa −65 °C. [Ag{Ni(CO)_4_(CH_2_Cl_2_)_2_}]^+^ and Ni(CO)_4_ have been calculated at the B3LYP-D3(BJ)/def2-TZVPP level. Frequencies are in cm^−1^. Calculated frequencies are scaled by 0.968 according to Duncan *et al.*^56^

Compound	IR bands: *ṽ*(CO)
[Ag{Ni(CO)_4_(CH_2_Cl_2_)_2_}]^+^ exp.	2151, 2106, 2074, 2050
[Ag{Ni(CO)_4_(CH_2_Cl_2_)_2_}]^+^ calc.	2152, 2110, 2084, 2074
Ni(CO)_4_ exp.	2057
Ni(CO)_4_ calc.	2051

The bonding situation was further investigated using energy decomposition analyses and extended transition-state analyses with natural orbitals for chemical valence (ETS-NOCV).^57,58^ The results are shown in Table 3. To account for the effects of the crystal environment, analyses were performed using both the optimized structure at the B3LYP-D3(BJ)/def2-TZVPP level and the crystal structure. Dielectric effects of the crystal environment were estimated using the COSMO model. However, both effects have only a minor impact on the results: Pauli repulsion and electrostatic interaction offset almost exactly, leaving a sizable and stabilizing orbital interaction term, that can be partly compensated by dielectric contributions of the environment. The orbital interaction between both fragments is dominated by the interaction between the HOMO of Ni(CO)_4_ (which is of d_*z*^2^_ nature) and the 5s-like LUMO of the Ag^+^ fragment (see Table S5 in the SI), as can be expected for a Lewis acid–base interaction. This interaction gives about 55% of the overall orbital interaction. However, there are additional, albeit weaker, interactions, which account for about 15% of the overall orbital contributions. They can be characterized as π-backbonding interactions from filled Ag 4d-orbitals (of d_*xz*_/d_*yz*_ type) into the π* orbitals of the CO ligands. These interactions are most likely the origin of the bending of the CO units observed in the crystal structure.

**Table 3:** Results of energy decomposition analysis of the interaction between Ni(CO)_4_ and [Ag(CH_2_Cl_2_)_2_]^+^ using both the crystal structure (crystal) and the quantum-chemically optimized structure (optimized at B3LYP-D3(BJ)/def2-TZVPP level) at BP86-D4/TZ2P level of theory in kJ mol^−1^

Structure	Δ*E*_Pauli_	Δ*E*_Elstat._	Δ*E*_Orb._	Δ*E*_COSMO_^a^	Δ*E*_Int_
Crystal	245.7	−194.5	−145.1	—	−133.8
Crystal b	242.2	−190.5	−146.3	37.5	−96.8
Optimized	210.0	−164.8	−131.0	—	−125.7
Optimized b	206.1	−159.4	−133.7	35.9	−91.1

a Contribution only includes the electrostatic interaction energy with the cavity charges.

b Calculations included the COSMO model with the parametrization for CH_2_Cl_2_ to approximate dielectric effects of the crystal environment.

## Conclusions

To conclude, we present a facile one-pot synthesis with high yields, requiring little to no purification, allowing access to the storable and highly electrophilic silver salt Ag[µ-(CN)(BCF)_2_] (proposed abbreviation Ag[BCNB]) as CH_2_Cl_2_ or *o*DFB solvate. The weak coordination ability of the [BCNB] anion was exploited to further react Ag[BCNB] with Ni(CO)_4_ forming the first structurally characterized coordination compound featuring Ni(CO)_4_ as a metalloligand. The resulting [Ag{Ni(CO)_4_}(CH_2_Cl_2_)_2_][BCNB] is, however, highly unstable, already decomposing at low temperatures (*ca.* −40 °C) and when exposed to reduced pressure. Nevertheless, we are convinced that the excellent properties of the [BCNB] anion, such as very low basicity, good solubility and structural rigidity will enable the synthesis of other highly electrophilic cations in the future and revive the interest in this weakly-coordinating anion. Consequently, the easy accessibility of Ag[BCNB] and its potential versatility as a Lewis acid, for halide abstractions as well as an oxidant could make it an attractive alternative to hitherto used Ag-WCA salts.

## Author contributions

W. R. B. and A. L. M. performed synthetic work and formal data analysis. M. R. performed quantum chemical calculations. R. S. and T.-N. S. collected XRD data. W. R. B. and S. M. R. refined XRD data. W. R. B. and M. R. wrote the manuscript (original draft). W. R. B., M. R., M. K. and M. M. revised the manuscript. M. M. conceptualised, coordinated and supervised the project.

## Conflicts of interest

There are no conflicts to declare.

## Supplementary Material

SC-017-D5SC05589J-s001

SC-017-D5SC05589J-s002

## Data Availability

CCDC 2471678 and 2471679 contain the supplementary crystallographic data for this paper.^59*a*,*b*^ Data supporting this manuscript is available within the supplementary information (SI) and available on request. Supplementary information: NMR, vibrational spectra and crystallographic data. See DOI: https://doi.org/10.1039/d5sc05589j.
