# Supplementary material for: A facile route to ‘naked’ Ag+ ions enabling the coordination of the weak Lewis base Ni(CO)4
Source: Chem Sci. 2025 Nov 7;17(1):406–11. doi: 10.1039/d5sc05589j (PMC12613210; doi:10.1039/d5sc05589j)
Supplement: SC-017-D5SC05589J-s001 [file SC-017-D5SC05589J-s001.pdf]

Supplementary Information (SI) for Chemical Science.
This journal is © The Royal Society of Chemistry 2025

Supporting information for

A facile route to 'naked' Ag^+ ions enabling the coordination of the weak Lewis base $Ni(CO)_4$

Willi R. Berg,[a] Amina L. Moshtaha,[a] Robin Sievers,[a] Marc Reimann,[b] Tim-Niclas Streit,[a] Susanne M. Rupf,[a], Martin Kaupp[b] and Moritz Malischewski*[a]

[a] W. R. Berg, A. L. Moshtaha, T.-N. Streit, R. Sievers, Dr. S. M. Rupf, Dr. M. Malischewski*
Institut für Chemie und Biochemie, Anorganische Chemie
Freie Universität Berlin
Fabeckstraße 34–36, 14195 Berlin (Germany)
E-mail: moritz.malischewski@fu-berlin.de

[b] Dr. M. Reimann, Prof. Dr. M. Kaupp
Institut für Chemie, Theoretische Chemie/Quantenchemie, Sekr. C7
Technische Universität Berlin
Straße des 17. Juni 135, 10623 Berlin (Germany)

[c] Dr. M. Reimann
Institut für Ionenphysik und Angewandte Physik
Universität Innsbruck,
Technikerstr. 25/3, 6020 Innsbruck, Austria

Experimental details

All experiments were performed under rigorous exclusion of moisture and oxygen using standard Schlenk techniques. $Ni(CO)_4$ is a highly toxic and flammable material and should only be handled by trained personnel with suitable protective equipment. Being stored in a gas cylinder, small amounts were condensed into the reaction vessel via vacuum transfer.

Solids were handled in an argon-filled glovebox, which was also equipped with a Bruker ALPHA FTIR spectrometer with a diamond ATR attachment. Raman spectra were recorded on a Bruker MultiRAM II equipped with a low-temperature Ge detector (1064 nm). Characteristic absorptions are given in wavenumbers $\tilde{\nu}$ [cm^{-1}] and intensities are stated as vs (very strong), s (strong), m (medium) and w (weak), vw (very weak), sh. (shoulder). The software OriginPro 2017G was used to plot the data.[1]

Low-temperature IR

IR spectra of single crystals were recorded on a Nicolet iS50 Advance FTIR by Thermo Fisher Scientific equipped with an ATR unit, with a Ge on KBr beamsplitter and a DLaTGS-KBr detector for MIR and a solid-substrate beamsplitter with a DLaTGS-PE detector for FIR. For low-temperature measurements we used a metal cylinder cooled by a cold N_2 stream (see Figures S1 and S2 for details).

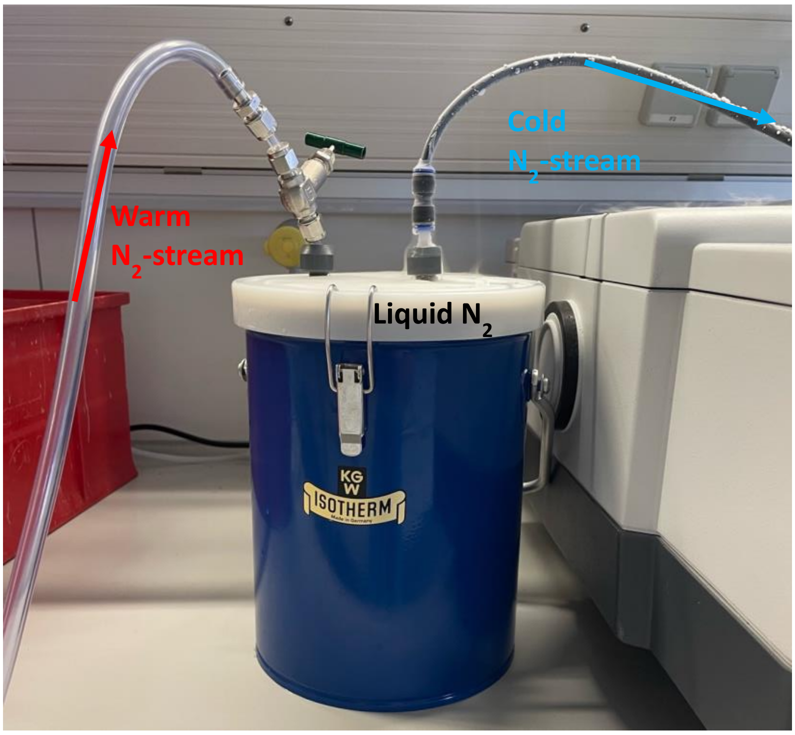

Figure S1. Experimental set-up for low-temperature IR measurements. Warm N_2-stream (red) runs through a dewar filled with liquid N_2 (–196 °C) generating a cold N_2 stream (light blue).

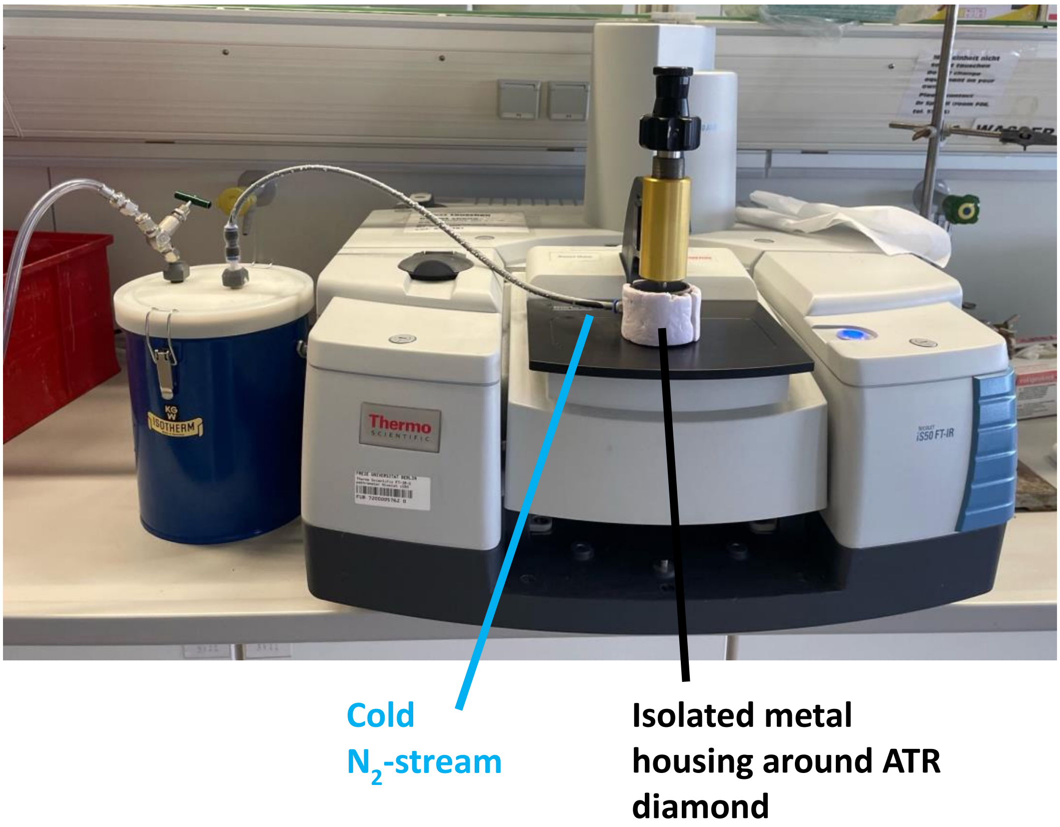

Figure S2. Experimental set-up for low-temperature IR measurements. The cold N_2 stream (light blue) is directed to an isolated metal housing around the ATR diamond on which the crystalline $[Ag\{Ni(CO)_4\}(CH_2Cl_2)_2][\mu\text{-}(CN)(BCF)_2]$ is placed.

NMR

NMR spectra were measured on a JEOL ECX 400 (400 MHz) in CD_2Cl_2 or CD_3CN. All spectra are device-internally calibrated. The given multiplicities are phenomenological, thus the actual appearance of the signals is stated and not the theoretically expected one. The following abbreviations were used and analogously combined to designate multiplicities: s (singlet), br (broad). For centrosymmetric multiplets the center and for non-symmetric multiplets the interval is stated. Evaluation of spectra was performed with Mestrelab Research MNova 7.[2]

X-Ray Diffraction (XRD)

X-Ray data was measured on a BRUKER X8 KAPPA APEX II diffractometer. The data was collected at 100-105 K using graphite-monochromated Mo Kα radiation ($\lambda\alpha$ = 0.71073 Å). The strategy for the data collected on was evaluated by using the Smart software. The strategy for the data collection was gathered by the standard "ψ-ω scan techniques" and were scaled and reduced using Saint+ software. The structures were solved by using Olex2[3] and XT[4] structure solution program using Intrinsic Phasing and refined with the XL refinement package[5] using at Least Squares minimization. For $[Ag\{Ni(CO)_4\}(CH_2Cl_2)_2][\mu\text{-}(CN)(BCF)_2]$, the crystal represents a solid solution of ca. 90% $[Ag\{Ni(CO)_4\}(CH_2Cl_2)_2][\mu\text{-}(CN)(BCF)_2]$ and 10% $[Ag(CH_2Cl_2)_3][\mu\text{-}(CN)(BCF)_2]$. All disordered positions have been treated through refinement of fractional occupancies, using a second free variable to define the fraction of disordered sites.

Additionally, the positional disorder of CH_2Cl_2 ligands was modelled with restraints (DFIX) and constraints (EADP) while the substitutional disorder of the [µ-(CN)(BCF)$_2$] anion was refined with constraints (EADP, EXYZ) to fix the thermal displacement parameters of N1, N014, C016 and C39. Crystal drawings were generated with Diamond[6] and POVRay.[7]

Experimental part:

Ag[µ-(CN)(BCF)$_2$] – "Ag[BCNB]"

General considerations

The stoichiometry of these smaller-scale reactions is sometimes difficult to achieve, and deviations will be visible in the product NMR spectra. Using too much AgCN will result in the presence of the monoadducts [CN–BCF]$^-$ and [NC–BCF]$^-$. The ^{19}F and ^{11}B NMR signals can be seen in Figures S7, S8, S13 and S14. (The nitrogen-bound boron signal of [CN–BCF]$^-$ is harder to recognize in the ^{11}B NMR because of the really broad signals.) Using too little AgCN will result in free BCF that can simply be removed by washing with *n*-pentane. However, an excess of BCF could be a disadvantageous due to the high water sensitivity of the free Lewis acid. Traces of water lead to the formation of BCF•H_2O, which then degrades to C_6F_5H, $(F_5C_6)_2BOH$ and $(F_5C_6)_2BOB(C_6F_5)_2$, yielding multiple impurities that are visible in the NMR spectra.

Complete conversion can generally be ensured by reaction control *via* ^{19}F NMR spectroscopy by diluting 0.2 mL of the reaction solution with additional *o*DFB or CH_2Cl_2. If too little AgCN is used and monoadducts are visible during the reaction control, additional BCF can easily be added.

Ag[µ-(CN)(BCF)$_2$] is stored at rt under an inert atmosphere and under exclusion of light.

The reaction solvents, CH_2Cl_2 and *o*DFB, remain coordinated to the silver salt after product is worked up and dried. The amount of coordinated solvent varies by batch but is approximately 1.3 molecules of CH_2Cl_2 or 1.5 to 2 molecules of *o*DFB. Harsher drying for a longer period of time or at elevated temperatures should be avoided, because BCF can evaporate or sublimate under these conditions, resulting in the degradation of the anion [µ-(CN)(BCF)$_2$] (Figure S7, S8, S13 and S14).

In CH_2Cl_2:

Ag(CN) (1.00 eq., 26.00 mg, 0.194 mmol) and BCF (2.02 eq., 200 mg, 0.391 mmol) are stirred in CH_2Cl_2 (4 mL) for 1 ½ h at rt resulting in a clear solution. The solvent is removed under reduced pressure. The resulting solid is washed with *n*-pentane (3 x 1 mL) and dried under reduced pressure. Ag[µ-(CN)(BCF)$_2$]•1.3 CH_2Cl_2 is obtained as colorless solid (202 mg, 0.159 mmol, 82%).

After washing and drying, Ag[µ-(CN)(BCF)$_2$] still contains a significant amount of CH_2Cl_2 that coordinates to Ag^+. This can be seen in both, the IR-spectrum and the ^{1}H NMR spectrum measured in CD_3CN (Figure S6 and S10). The compound appears to partially degrade in CD_3CN, resulting in impurities. *o*DFB was added to the NMR sample in CD_3CN as a reference. Comparing the ^{19}F NMR integrals of [µ-(CN)(BCF)$_2$] and *o*DFB to the ^{1}H NMR signals of *o*DFB and CH_2Cl_2 indicates that approximately 1.3 molecules of CH_2Cl_2 remain coordinated to Ag[µ-(CN)(BCF)$_2$] after the work-up (Figure S5 and S6).

It was investigated whether longer drying (15 h) at room temperature or drying at an elevated temperature (50 °C) could remove the remaining solvent. However, we found that CH_2Cl_2 remains coordinated, while Ag[μ-(CN)(BCF)$_2$] partially degrades under these conditions. The coordinated BCF is removed from [μ-(CN)(BCF)$_2$]$^-$ upon longer exposure times or elevated temperatures under reduced pressure, yielding the monoadducts CN–BCF and NC–BCF (Figure S8 and S9). The NMR spectra after drying were measured solely in CD_2Cl_2 instead of CD_3CN to avoid further degradation and demonstrate the decomposition caused by harsher drying attempts. Even after drying, the ^{1}H NMR spectra still show a substantial amount of CH_2Cl_2 present beside the $CDHCl_2$ signal (Figure S9).

^{11}B NMR (128 MHz, CD_2Cl_2, rt): δ [ppm] = –13 (br, N–B), –23 (br, C–B).

^{19}F NMR (377 MHz, CD_2Cl_2, rt): δ [ppm] = –133.42 (d, $J_{F–F}$ = 22.6 Hz, 6 F, *o*-F), –134.54 (d, $J_{F–F}$ = 18.9 Hz, 6 F, *o*-F), –159.13 (t, $J_{F–F}$ = 20.6 Hz, 3 F, *p*-F), –159.29 (t, $J_{F–F}$ = 20.6 Hz, 3 F, *p*-F), –165.54 (m, 6 F, *m*-F), –165.86 (m, 6 F, *m*-F).

IR (ATR, bulk, rt): $\tilde{\nu}$ [cm^{-1}] = 2290 (w, ν(CN), 1646 (m), 1516 (s), 1459 (vs), 1382 (m), 1338 (sh), 1284 (m), 1258 (sh), 1179 (vw), 1095 (s), 1029 (vw), 1006 (sh), 973 (vs), 935 (sh), 902 (m), 880 (w), 812 (m), 768 (m), 719 (m), 683 (s), 675 (sh), 627 (w), 609 (w), 575 (w), 482 (vw), 456 (vw), 440 (vw).

The spectroscopic data of [μ-(CN)(BCF)$_2$]$^-$ is in accordance with the literature.[8]

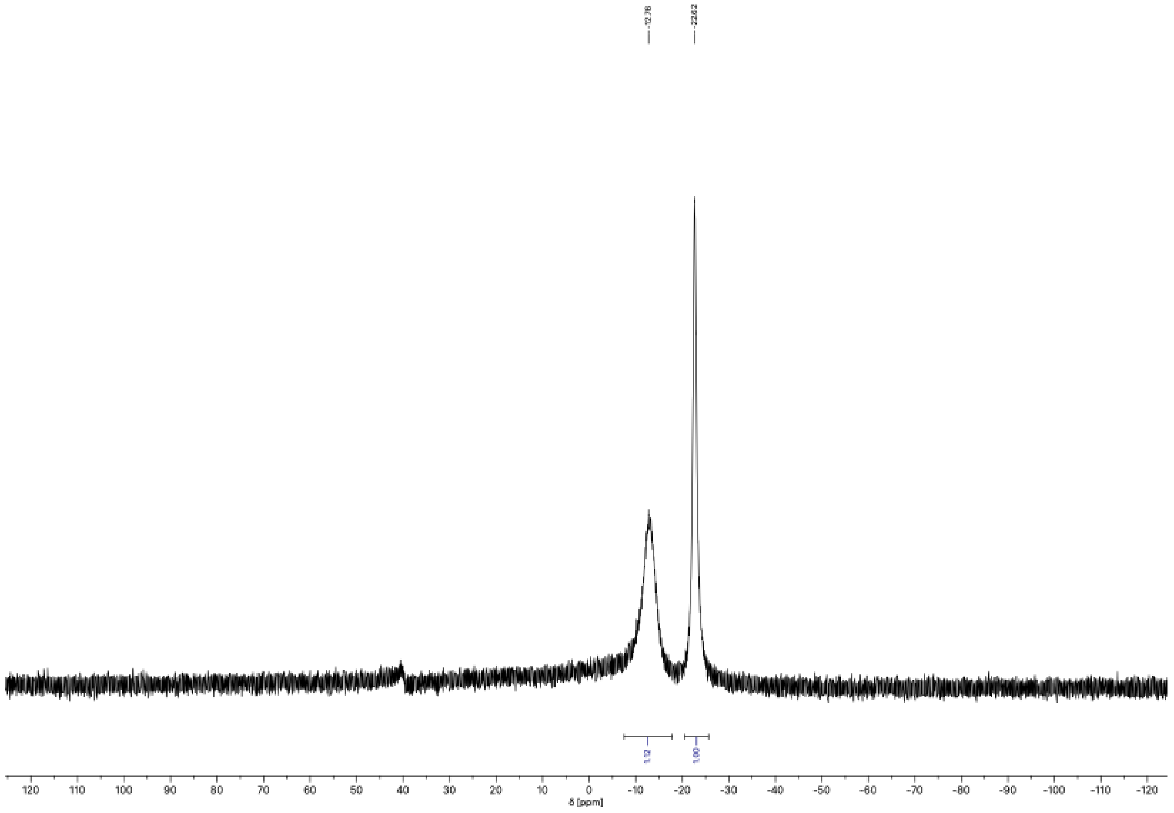

Figure S3. ^{11}B NMR spectrum (128 MHz, CD_2Cl_2, rt) of Ag[μ-(CN)(BCF)$_2$] synthesized in CH_2Cl_2.

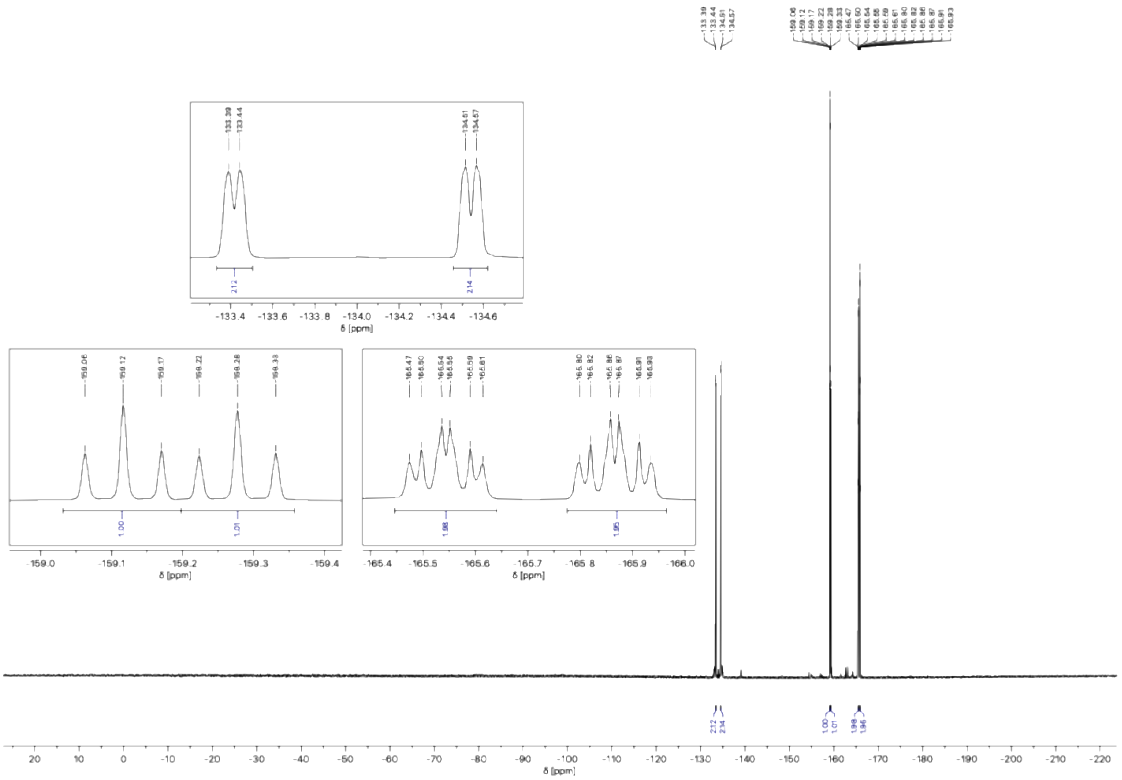

Figure S4. ^{19}F NMR spectrum (377 MHz, CD_2Cl_2, rt) of Ag[µ-(CN)(BCF)$_2$] synthesized in CH_2Cl_2.

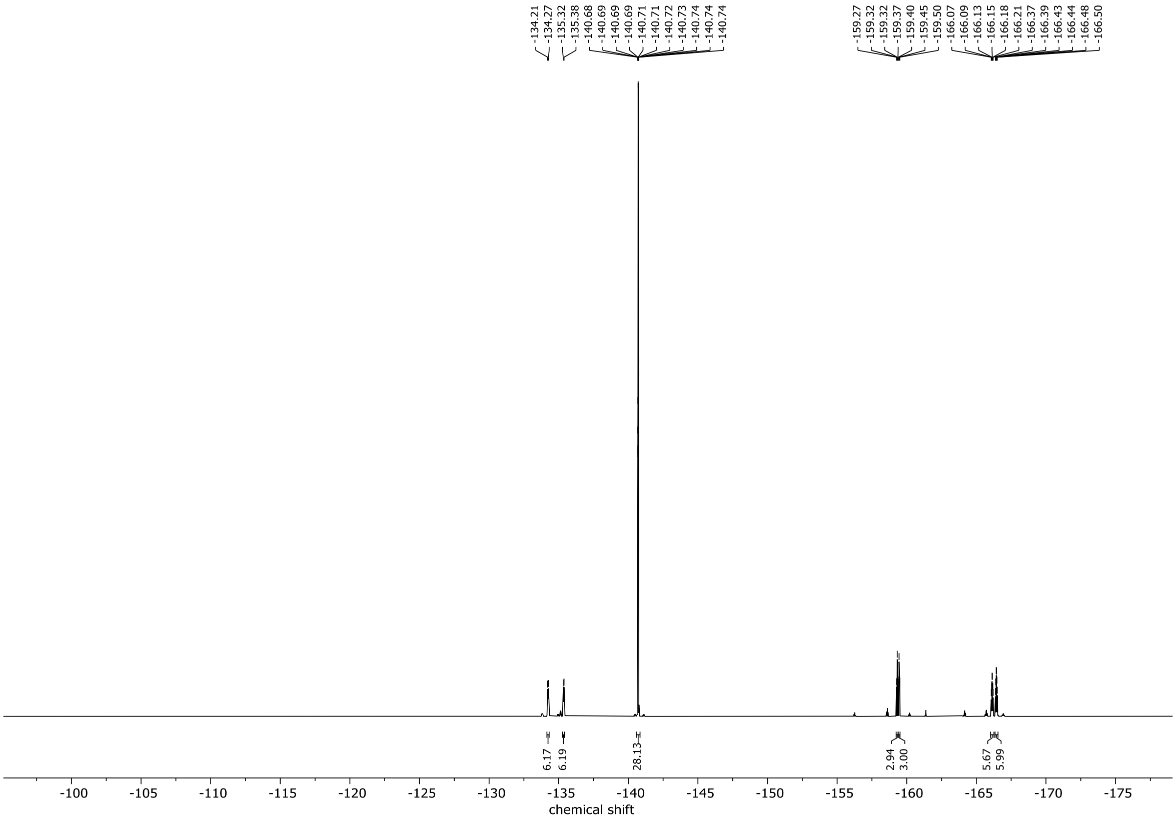

Figure S5. ^{19}F NMR spectrum (377 MHz, CD_3CN, rt) of Ag[µ-(CN)(BCF)$_2$] synthesized in CH_2Cl_2 with *o*DFB as reference.

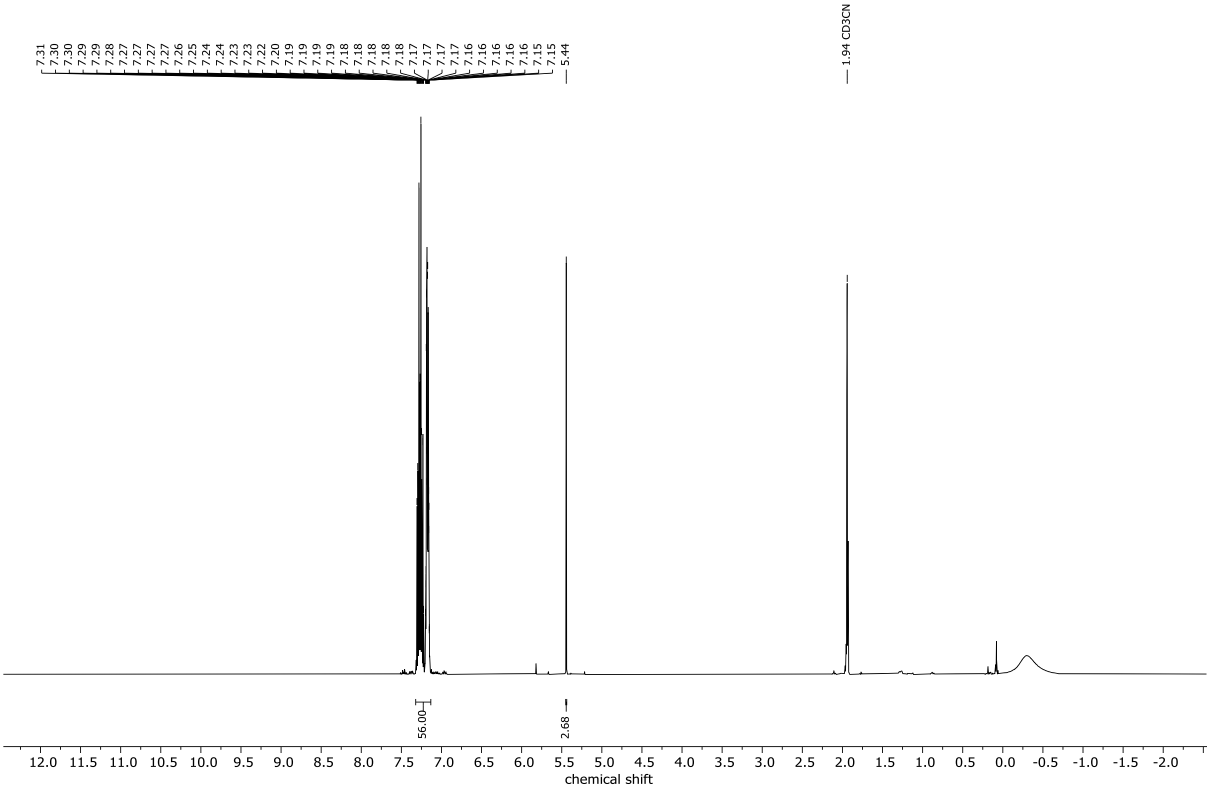

Figure S6. ^{1}H NMR spectrum (400 MHz, CD_3CN, rt) of Ag[μ-(CN)$(BCF)_2$] synthesized in CH_2Cl_2 with *o*DFB as reference.

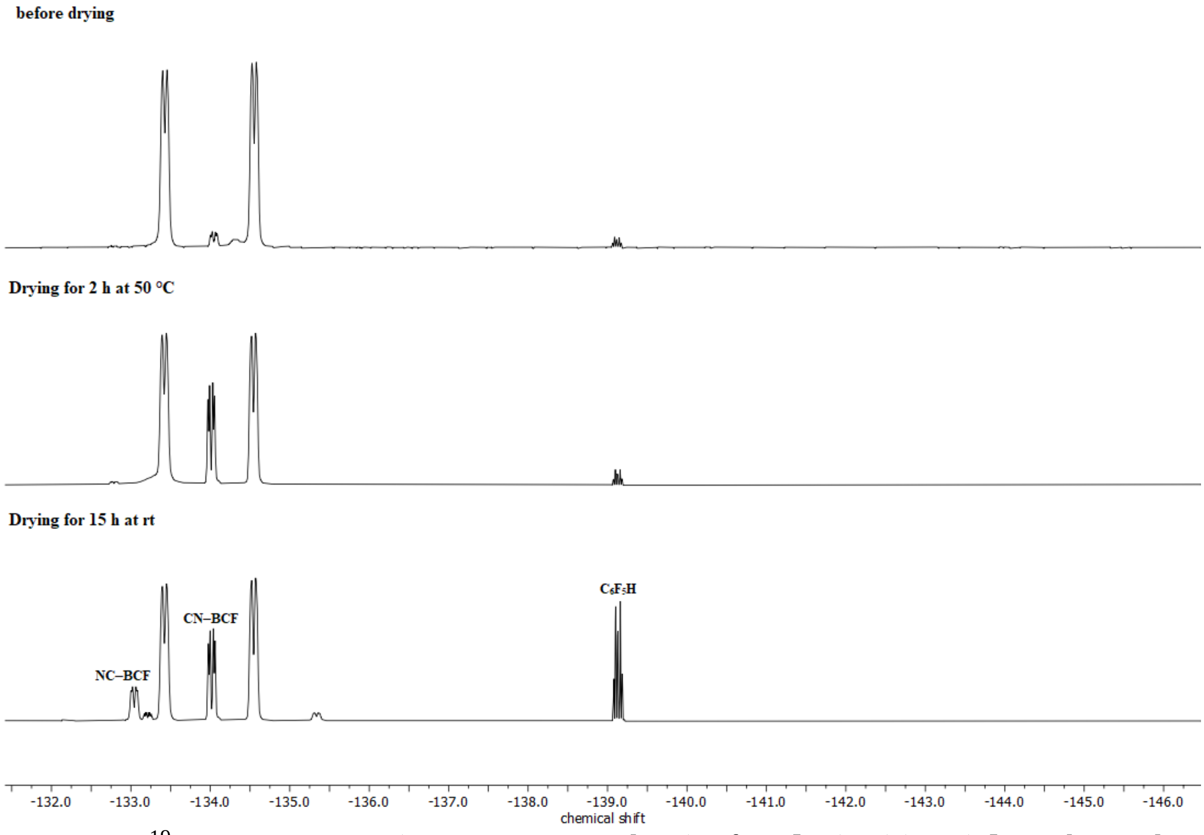

Figure S7. ^{19}F NMR spectra (377 MHz, CD_2Cl_2, rt) of Ag[μ-(CN)$(BCF)_2$] synthesized in CH_2Cl_2 before drying, after drying at 50 °C and after drying at rt with the degradation products.

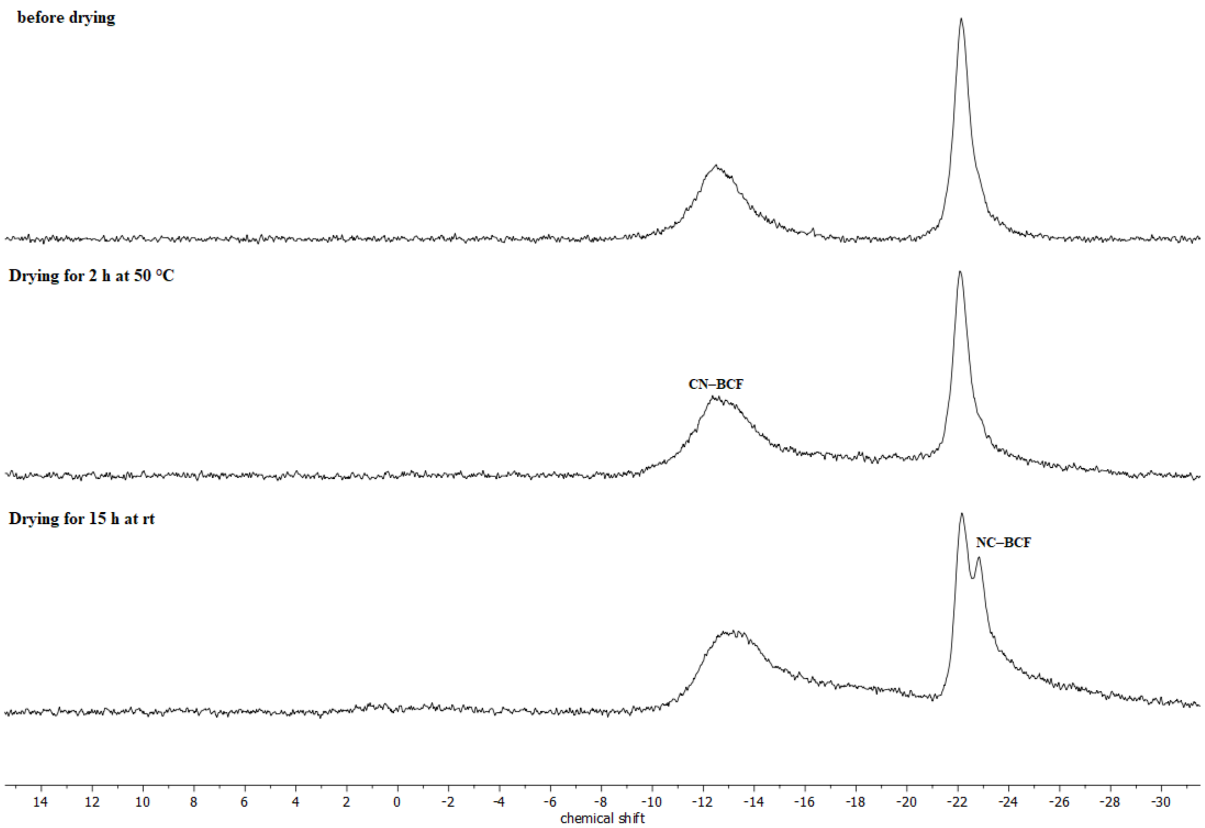

Figure S8. ^{11}B NMR spectra (128 MHz, CD_2Cl_2, rt) of Ag[μ-(CN)(BCF)$_2$] synthesized in CH_2Cl_2 before drying, after drying at 50 °C and after drying at rt with the degradation products.

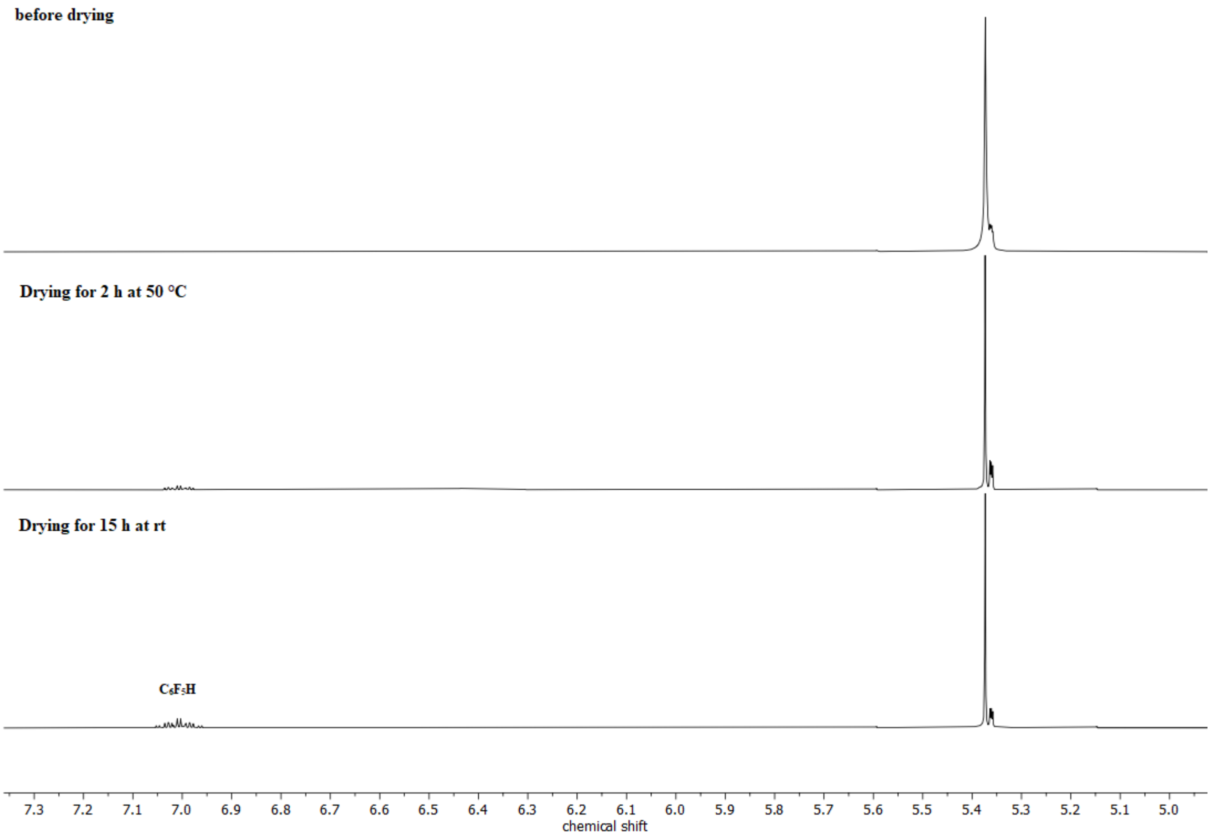

Figure S9. ^{1}H NMR spectra (400 MHz, CD_2Cl_2, rt) of Ag[μ-(CN)(BCF)$_2$] synthesized in CH_2Cl_2 before drying, after drying at 50 °C and after drying at rt with the degradation products.

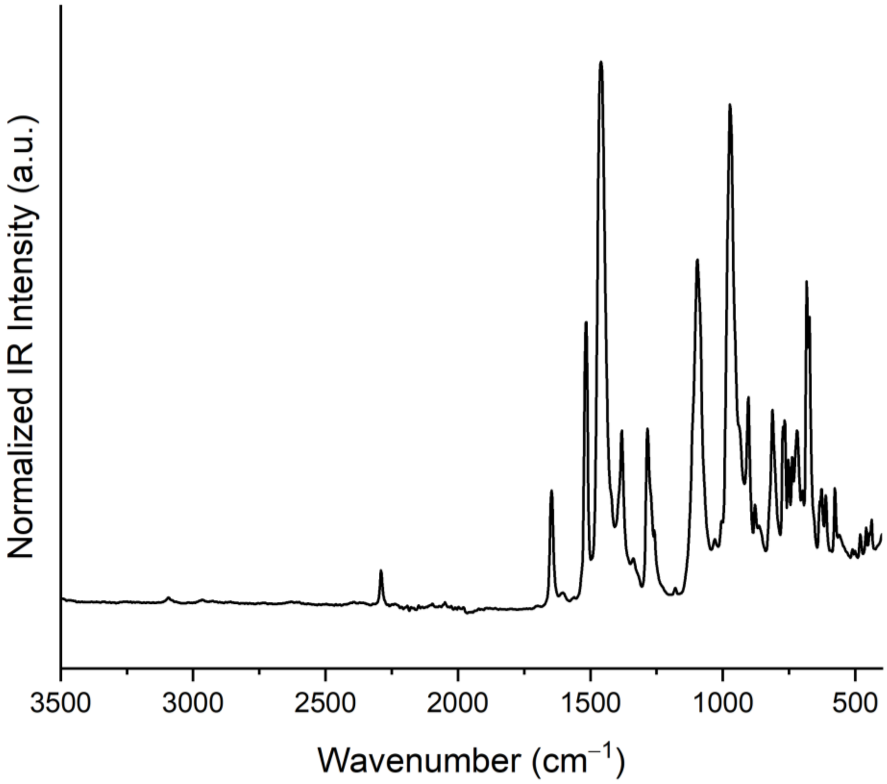

Figure S10. IR spectrum (ATR, bulk, rt) of Ag[µ-(CN)(BCF)$_2$] synthesized in CH_2Cl_2.

In *o*DFB:

Ag(CN) (1.0 eq., 26.0 mg, 0.194 mmol) and BCF (2.02 eq., 200 mg, 0.391 mmol) are stirred in *o*DFB (4 mL) for 2 h at rt resulting in a clear solution. The solvent is removed under reduced pressure. The resulting solid is washed with *n*-pentane (3 x 1 mL) and dried under reduced pressure. Ag[µ-(CN)(BCF)$_2$]•1.45 *o*DFB is obtained as colorless solid (240 mg, 0.181 mmol, 93%).
Single crystals are obtained by layering the reaction mixture with *n*-pentane, followed by cooling to –24 °C.

After washing and drying, Ag[µ-(CN)(BCF)$_2$] still contains a significant amount of *o*DFB that coordinates to Ag^+. This can be seen in both, the IR-spectrum and in the ^{1}H and ^{19}F NMR spectra. The amount of *o*DFB molecules coordinating per Ag^+ was determined to be 1.45 molecules *via* fluorine integrals in the ^{19}F NMR spectrum. This value was considered in the calculation of the yield. As with the product obtained from CH_2Cl_2, it was investigated whether longer drying (20 h) at room temperature or drying at an elevated temperature (50 °C) could remove the remaining solvent. However, it was shown that *o*DFB remains coordinated while Ag[µ-(CN)(BCF)$_2$] partially degrades under these conditions. The coordinated BCF is removed from [µ-(CN)(BCF)$_2$]$^-$ under reduced pressure at longer times or elevated temperatures, yielding the monoadducts CN–BCF and NC–BCF (Figure S13 and S14).

^{11}B NMR (128 MHz, CD_2Cl_2, rt): δ [ppm] = –15 (br, N–B), –24. (br, C–B).

^{19}F NMR (377 MHz, CD_2Cl_2, rt): δ [ppm] = –133.43 (d, $J_{F–F}$ = 22.8 Hz, 6 F, *o*-F), –134.55 (d, $J_{F–F}$ = 18.8 Hz, 6 F, *o*-F), –159.13 (t, $J_{F–F}$ = 20.2 Hz, 3 F, *p*-F), –159.29 (t, $J_{F–F}$ = 20.3 Hz, 3 F, *p*-F), –165.48 - –165.64 (m, 6 F, *m*-F), –165.78 - –165.98 (m, 6 F, *m*-F).

IR (ATR, bulk, rt): $\tilde{\nu}$ [cm^{-1}] = 2301 (w, *ν*(CN)), 1647 (m), 1598 (w), 1518 (s), 1506 (m), 1461 (vs), 1382 (m), 1320 (w), 1276 (m), 1217 (w), 1166 (vw), 1098 (s), 1012 (w), 974 (vs), 941 (sh), 910 (m), 878 (vw), 848 (w), 814 (w), 784 (m), 770 (m), 740 (m), 680 (s), 617 (w), 577 (w), 566 (w), 548 (w), 456 (vw).

The spectroscopic data of [μ-(CN)$(BCF)_2]^-$ is in accordance with the literature.[8]

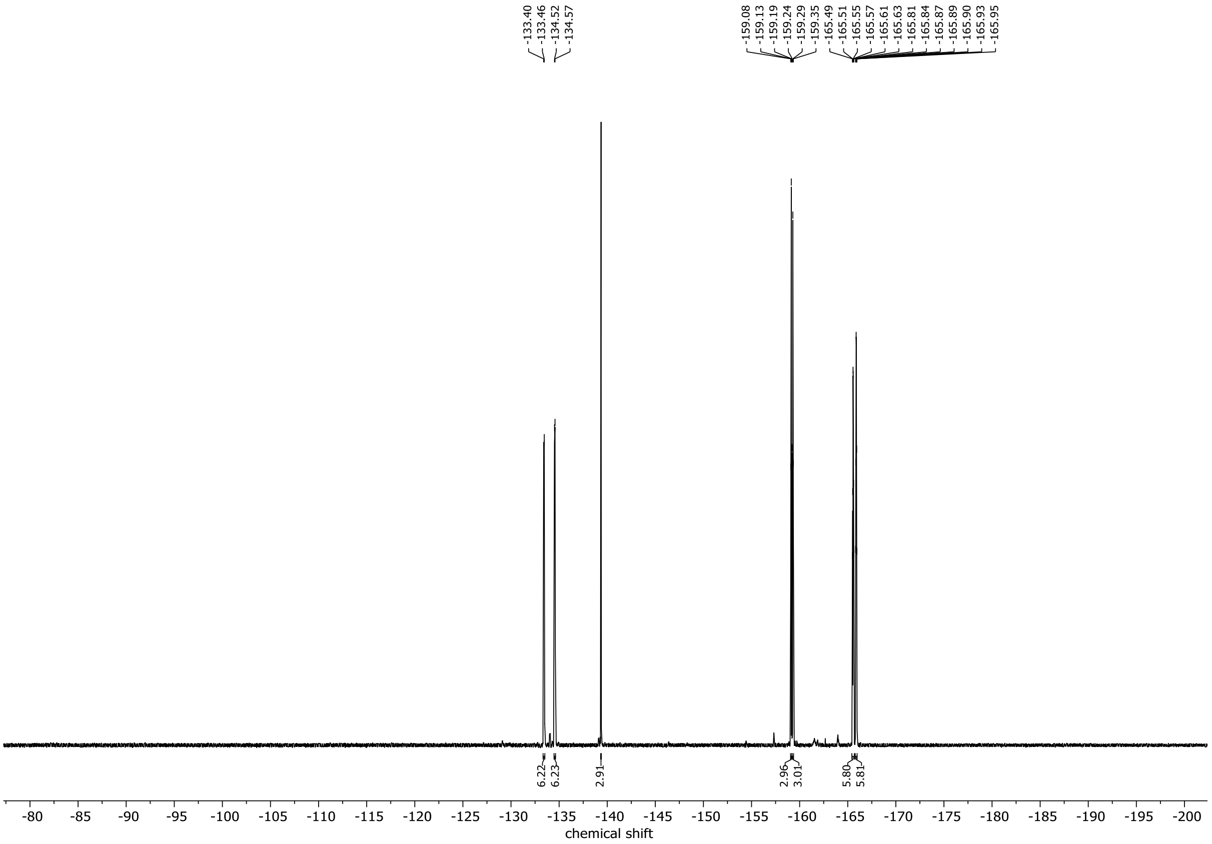

Figure S11. ^{19}F NMR spectrum (377 MHz, CD_2Cl_2, rt) of Ag[μ-(CN)$(BCF)_2$] synthesized in *o*DFB.

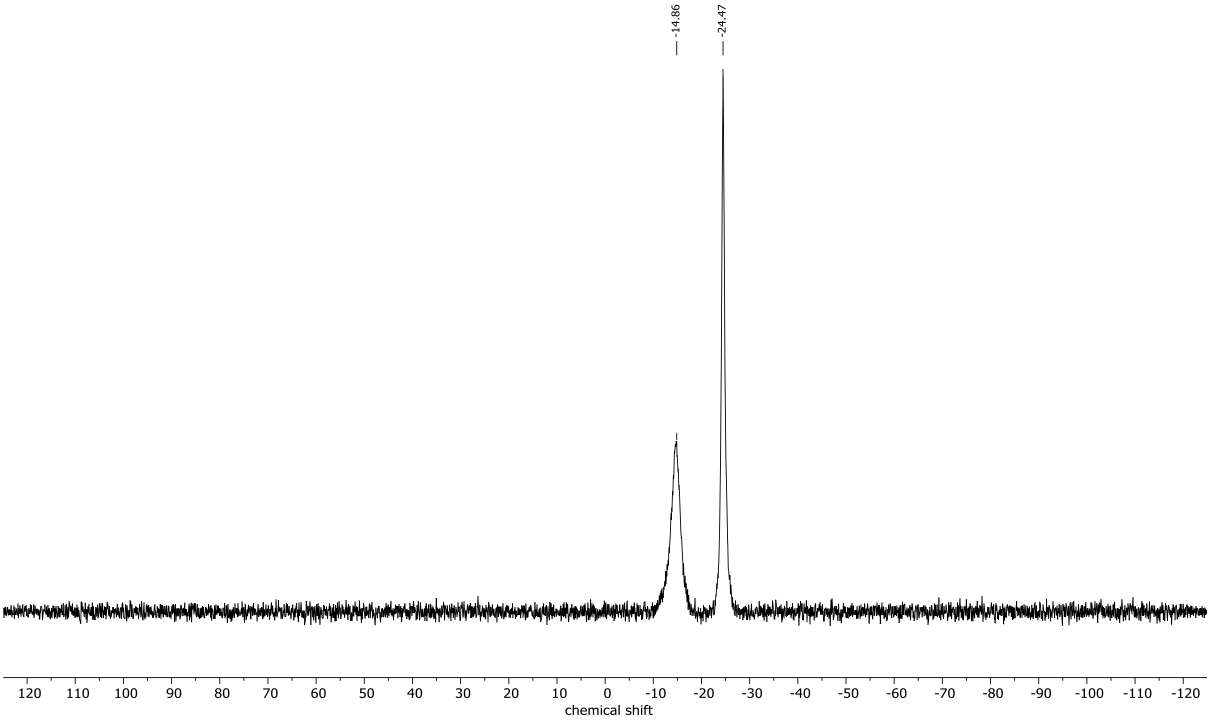

Figure S12. ^{11}B NMR spectrum (128 MHz, CD_2Cl_2, rt) of Ag[µ-(CN)(BCF)$_2$] synthesized in *o*DFB.

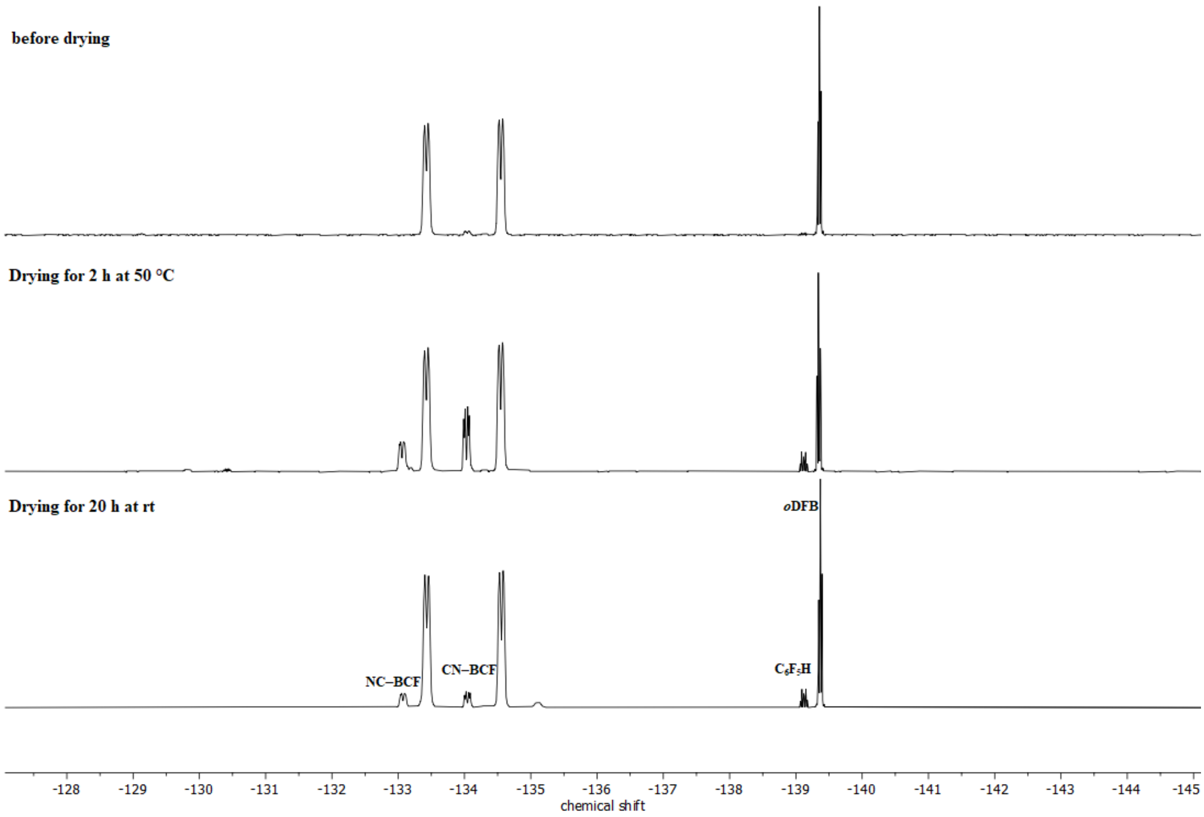

Figure S13. ^{19}F NMR spectra (377 MHz, CD_2Cl_2, rt) of Ag[µ-(CN)(BCF)$_2$] synthesized in *o*DFB before drying, after drying at 50 °C and after drying at rt with the degradation products.

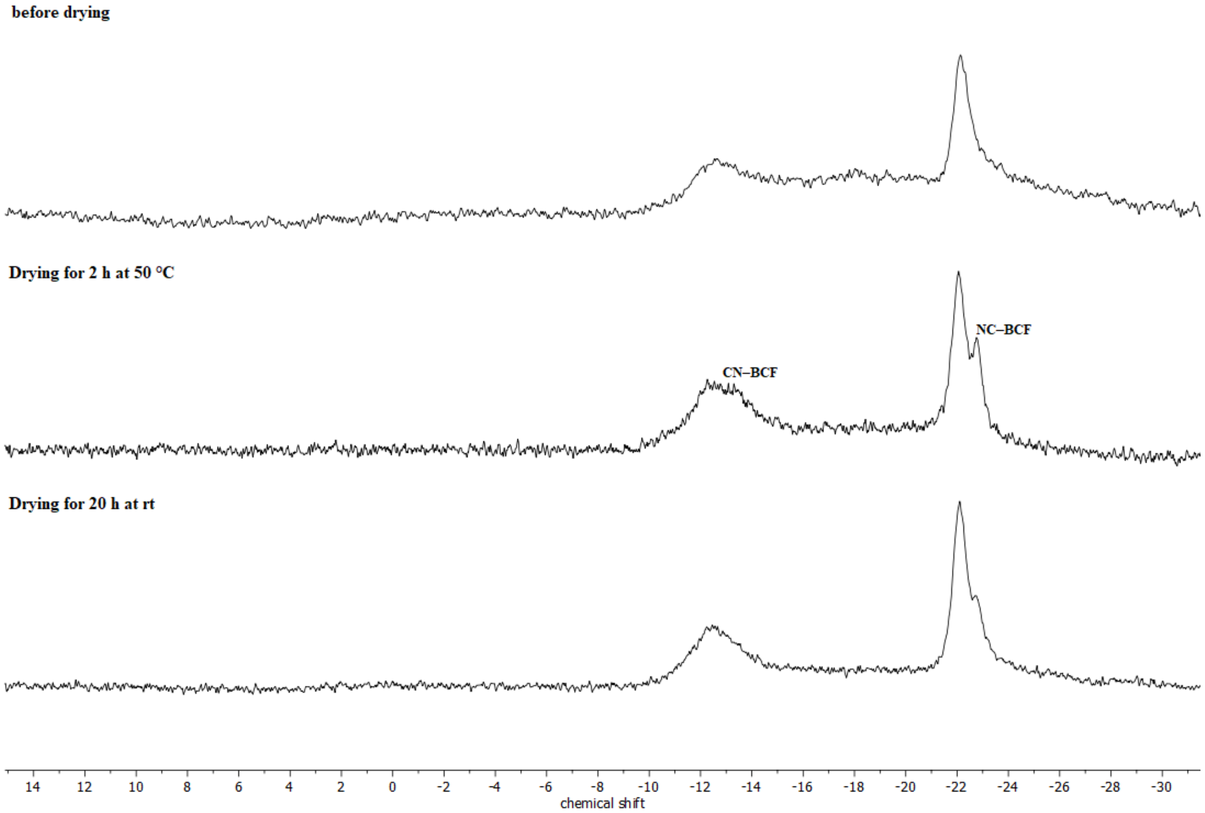

Figure S14. ^{11}B NMR spectra (128 MHz, CD_2Cl_2, rt) of Ag[μ-(CN)$(BCF)_2$] synthesized in *o*DFB before drying, after drying at 50 °C and after drying at rt with the degradation products.

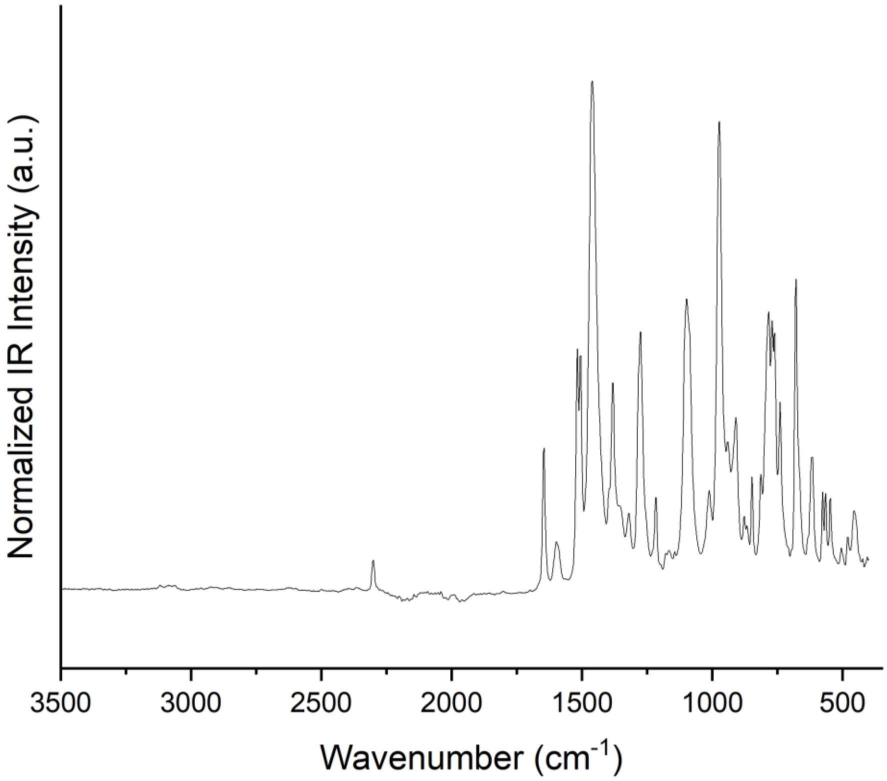

Figure S15. IR spectrum (ATR, bulk, rt) of Ag[μ-(CN)$(BCF)_2$] synthesized in *o*DFB.

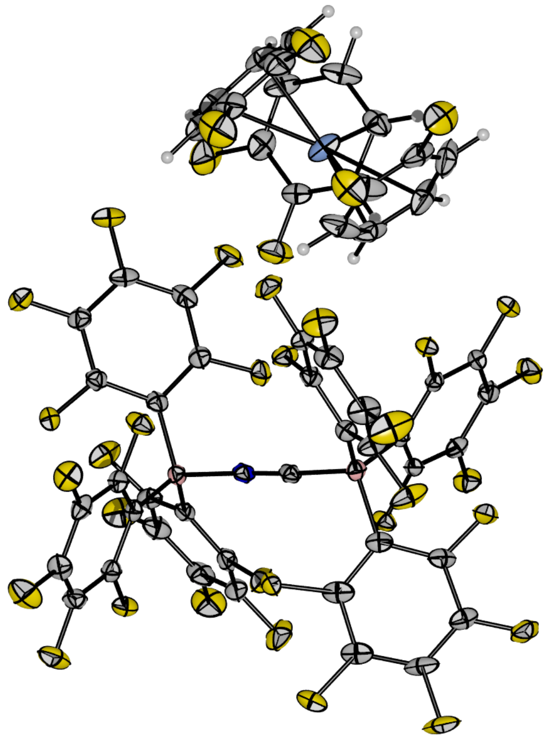

Figure S16. Molecular structure in the solid state of [Ag(oDFB)$_3$][μ-(CN)(BCF)$_2$]. Displacement ellipsoids are shown at the probability of 50%. Color code: light-blue=silver, dark-blue=nitrogen, grey=carbon, pink=boron, yellow=fluorine, white=hydrogen.

[Ag{Ni(CO)$_4$}(CH$_2$Cl$_2$)$_2$][BCF-CN-BCF]

In a 10 ml Schlenk tube Ag(CN) (1.00 eq., 13.0 mg, 0.0971 mmol) and BCF (2.02 eq., 100 mg, 0.195 mmol) are stirred in CH_2Cl_2 (1.5 mL) for 1 ½ h at rt resulting in a clear solution. $Ni(CO)_4$ (excess) was condensed on the frozen mixture at –196 °C. Subsequently, the reaction is warmed up to –60 °C and stirred at this temperature for 30 min. Colorless crystals suitable for single crystal X-ray diffraction were obtained by layering the reaction mixture with *n*-pentane (ca. 4 mL) at –60 °C and cooling to –70 °C over the course of several days.

IR (ATR, crystal, 233 K): $\tilde{\nu}$ [cm^{-1}] = 3074 (vw), 2299 (w, ν(CN)), 2151 (w, ν(CO)), 2106 (m, ν(CO)), 2074 (m, ν(CO)), 2050 (m, ν(CO)), 1651 (m), 1639 (sh), 1515 (s), 1461 (vs), 1395 (sh), 1382 (m), 1279 (m), 1265 (w), 1212 (w), 1168 (w), 1096 (m), 1082 (m), 1093 (w), 977 (s), 967 (sh), 951 (m), 911 (s), 883 (w), 817 (w), 764 (m), 738 (s), 680 (s), 627 (m), 606(w), 578 (w), 435 (m), 405 (s).

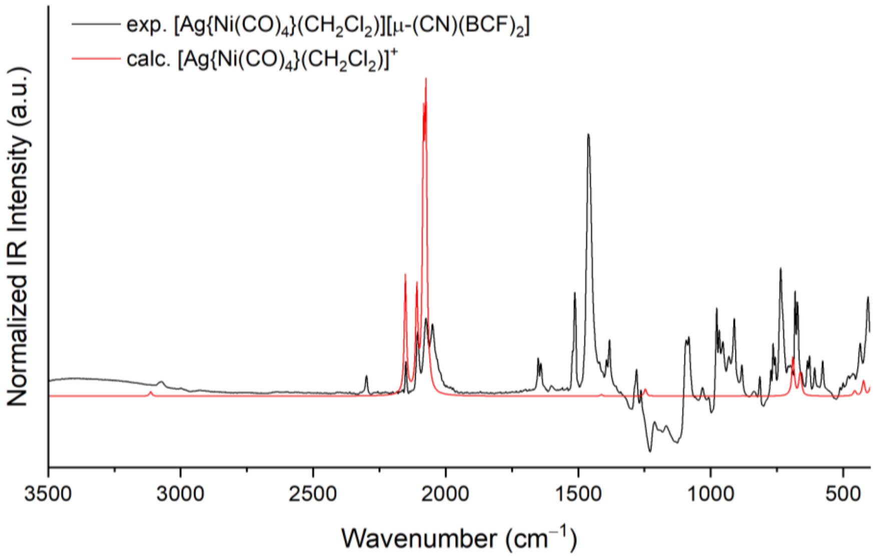

Figure S17. Low temperature IR (ATR, crystal, 233 K) spectrum of [Ag{Ni(CO)$_4$}(CH$_2$Cl$_2$)$_2$][µ-(CN)(BCF)$_2$] (black) compared to the calculated (B3LYP/def2-TZVPP) spectrum of [Ag{Ni(CO)$_4$}(CH$_2$Cl$_2$)$_2$]$^+$ (red).

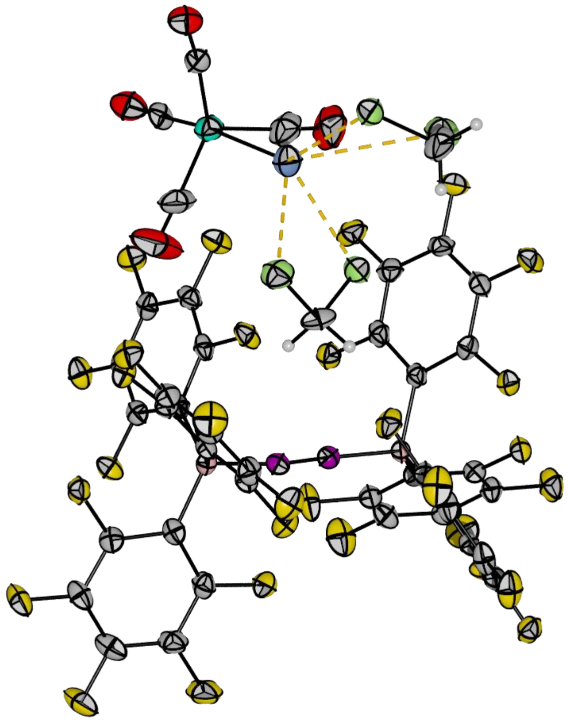

Figure S18. Solid-state structure of [Ag{Ni(CO)$_4$}(CH$_2$Cl$_2$)$_2$][μ-(CN)(BCF)$_2$] obtained from a co-crystal containing 90% [Ag{Ni(CO)$_4$}(CH$_2$Cl$_2$)$_2$][μ-(CN)(BCF)$_2$] and 10% [Ag(CH$_2$Cl$_2$)$_3$][μ-(CN)(BCF)$_2$]. Displacement ellipsoids are shown at the probability of 50%. Color code: blue=silver, turquoise=nickel, red=oxygen, grey=carbon, pink=boron, green=chlorine, yellow=fluorine, purple=carbon(50%)/nitrogen(50%) mixed site as cyanide bridging moiety, white=hydrogen. Selected bond lengths [pm] = Ag–Ni 258.6(6), Ag–Cl 255.7(2), 287.0(2), 289.3(2), 349.0(2), Ag–F 297.93 pm.

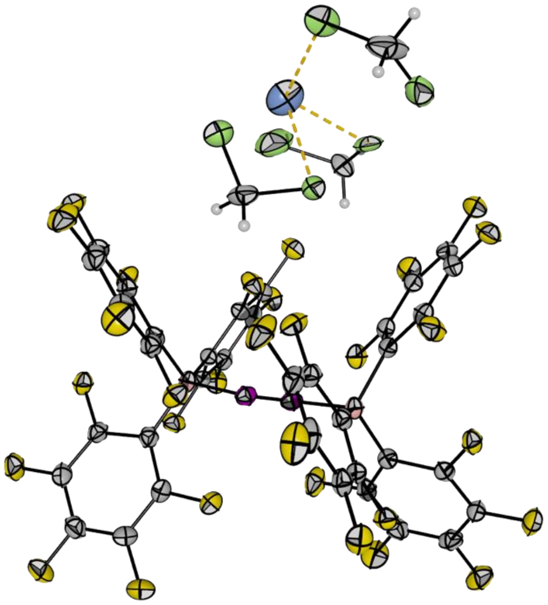

Figure S19. Solid-state structure of [Ag(CH$_2$Cl$_2$)$_3$][μ-(CN)(BCF)$_2$] obtained from a co-crystal containing 90% [Ag{Ni(CO)$_4$}(CH$_2$Cl$_2$)$_2$][μ-(CN)(BCF)$_2$] and 10% [Ag(CH$_2$Cl$_2$)$_3$][μ-(CN)(BCF)$_2$]. Displacement ellipsoids are shown at the probability of 50%. Color code:

blue=silver, grey=carbon, pink=boron, green=chlorine, yellow=fluorine, purple=carbon(50%)/nitrogen(50%) mixed site as cyanide bridging moiety, white=hydrogen.

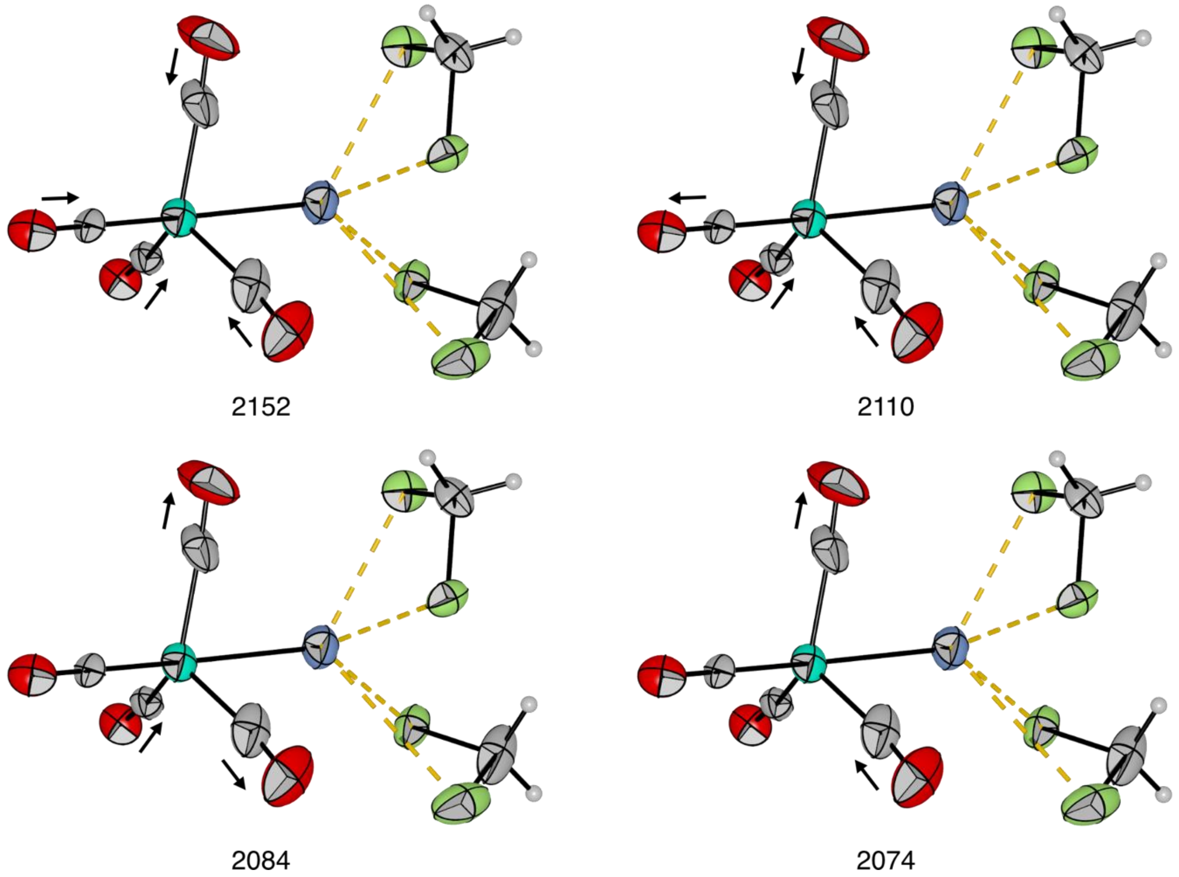

Figure S20. Vibrational analysis of the CO region in the IR spectrum (Figure S17) of crystalline $[Ag\{Ni(CO)_4\}(CH_2Cl_2)_2]^+$ shown is the molecular structure in the solid state of $[Ag\{Ni(CO)_4\}(CH_2Cl_2)_2]^+$ (Figure S18) with arrows besides the CO show the phase of the vibrational mode. Vibrational frequencies [cm^{-1}] correspond to calculated (B3LYP/def2-TZVPP) frequencies and are scaled by 0.968 according to Duncan *et al.* [9]

Quantum Chemical Calculations

Structure optimizations followed by harmonic vibrational frequency calculations were performed at the B3LYP-D3(BJ)/def2-TZVPP level of theory [10-12] using the Gaussian program (version 16 A.03).[13] All calculations employed default settings. Frequencies are scaled by 0.968 according to Duncan *et al.*[9]

Energy decomposition analyses (EDA) and extended transition-state analyses with natural orbitals for chemical valence (ETS-NOCV) were performed at the BP86-D4/TZ2P [14-17] level using the ADF engine of the AMS program package, release 2023.1. [18,19]

[Ag{Ni(CO)$_4$}(CH$_2$Cl$_2$)$_2$]$^+$

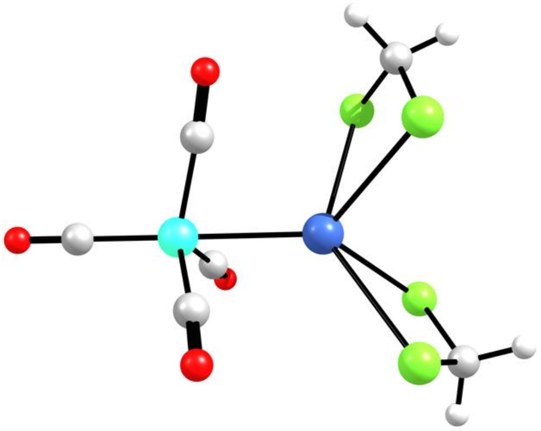

Figure S21. Calculated minimum structure of [Ag{Ni(CO)$_4$}(CH$_2$Cl$_2$)$_2$]$^+$ at B3LYP-D3(BJ)/def2-TZVPP level of theory. Color code: blue = silver, turquoise = nickel, grey = carbon, red = oxygen, green = chlorine, white = hydrogen.

Table S1. Calculated (B3LYP-D3(BJ)/def2-TZVPP) atomic coordinates of the minimum structure of [Ag{Ni(CO)$_4$}(CH$_2$Cl$_2$)$_2$]$^+$.

Ag	-0.479992000	-0.079756000	0.031555000
Ni	2.158093000	-0.164531000	-0.001810000
Cl	-1.994265000	1.648261000	-1.549222000
Cl	-2.533422000	-1.182985000	1.525742000
Cl	-1.280092000	2.596654000	1.158190000
Cl	-1.934333000	-2.351218000	-1.119762000
O	1.429441000	-0.705817000	2.838711000
O	5.132604000	-0.213568000	0.209686000
C	4.009884000	-0.196184000	0.131248000
O	1.602479000	-2.360408000	-1.958419000
C	1.625073000	-0.497578000	1.744354000
O	1.628621000	2.574640000	-1.067351000
C	-1.785424000	3.075299000	-0.479646000
H	-2.742314000	3.576325000	-0.412989000
H	-1.013841000	3.700018000	-0.910072000
C	1.769631000	-1.527302000	-1.214552000
C	1.785260000	1.529856000	-0.662473000
C	-3.038401000	-2.369787000	0.279166000
H	-3.008202000	-3.354542000	0.726823000
H	-4.028018000	-2.093108000	-0.059862000

Table S2. Calculated (B3LYP-D3(BJ)/def2TZVPP) IR frequencies of $[Ag\{Ni(CO)_4\}(CH_2Cl_2)_2]^+$. with their corresponding intensities. Frequencies are scaled by 0.968 according to Duncan *et al.*[9]

Wavenumber [cm^{-1}]	Intensity [KM/mol]
7.5	0.0
14.7	0.2
16.2	0.4
18.0	0.0
27.1	0.7
28.5	0.5
30.7	0.5
38.7	4.7
40.6	0.8
58.4	1.5
63.9	6.3
71.0	2.1
73.5	0.6
77.6	0.2
78.1	0.4
79.3	0.5
85.1	0.2
103.1	4.6
111.2	1.5
138.1	6.8
265.4	1.4
266.7	2.5
277.5	0.0
291.5	3.6
295.6	3.5
353.0	2.4
381.6	37.9
385.2	40.5
395.1	25.4
422.5	38.8
427.4	8.2
427.9	2.0
454.7	9.1
459.7	8.5
660.0	39.8
664.4	41.0
685.4	24.7
691.1	102.0
876.1	0.7
876.6	1.0
1124.8	0.0
1127.8	0.1
1242.8	1.4
1246.5	20.9

1412.3	4.3
1414.2	0.2
2074.4	772.8
2083.8	671.0
2109.7	294.3
2151.8	341.2
3030.6	0.3
3030.8	0.3
3113.6	6.8
3114.0	6.2

Comparison of bonding interactions of $Ni(CO)_4$ vs $Fe(CO)_5$ towards Ag^+

To compare the basicity of the metal carbonyls $Ni(CO)_4$ and $Fe(CO)_5$ towards Ag^+ the reaction enthalpy and Gibbs free energies in the gas phase were calculated. In a separate crystal structure which does not meet the requirements for publication, solvated $[Ag(CH_2Cl_2)_3]^+$ was obtained, similar to the *o*DFB adduct shown in Figure S16. Therefore, the ligand exchange of DCM against either metal carbonyl complexes was calculated.

Table S3. (Summed) eigenvalues of the most important contributions in an ETS-NOCV analysis of the interaction between $Ni(CO)_4$ and $[Ag(CH_2Cl_2)_2]^+$ using both the crystal structure (crystal) and the quantum-chemically optimized structure (optimized at B3LYP-D3(BJ)/def2-TZVPP level) at BP86-D4/TZ2P level in kJ/mol. Interactions shown are characterized as (donating orbital – accepting orbital).

structure	$Ni(CO)_4$ (d_{z^2}) – Ag^+ (5s)	Ag^+ ($d_{xz/yz}$) – $Ni(CO)_4$ (π^*)
crystal	−81.8	−22.9
crystal [a]	−78.2	−25.9
optimized	−74.6	−20.6
optimized [a]	−70.9	−24.7

[a]Calculations included the COSMO model with the parametrization for CH_2Cl_2 to approximate dielectric effects of the crystal environment.

Crystallographic Data

Table S4. Crystallographic data of [Ag(*o*DFB)$_3$][µ-(CN){B(C_6F_5)$_3$}$_2$].

Identification code	2471678
Empirical formula	$C_{55}H_{12}AgB_2F_{36}N$
Formula weight	1500.15
Temperature/K	100.00
Crystal system	triclinic
Space group	$P\bar{1}$
a/Å	11.4773(4)
b/Å	15.0019(7)
c/Å	17.3790(7)
α/°	111.9500(10)
β/°	96.586(2)
γ/°	109.0740(10)
Volume/Å^3	2525.48(18)
Z	2
ρ_{calc}g/cm^3	1.973
μ/mm^{-1}	0.577
F(000)	1460.0
Crystal size/mm^3	0.1 × 0.1 × 0.1
Radiation	MoKα (λ = 0.71073)
2Θ range for data collection/°	3.9 to 50.722
Index ranges	$-13 \leq h \leq 13$, $-18 \leq k \leq 18$, $-20 \leq l \leq 20$
Reflections collected	48637
Independent reflections	9238 [R_{int} = 0.0368, R_{sigma} = 0.0266]
Data/restraints/parameters	9238/5/938
Goodness-of-fit on F^2	1.085
Final R indexes [I>=2σ (I)]	R_1 = 0.0318, wR_2 = 0.0745
Final R indexes [all data]	R_1 = 0.0388, wR_2 = 0.0805
Largest diff. peak/hole / e Å^{-3}	0.37/-1.02

Table S5. Crystallographic data of co-crystallized $[Ag\{Ni(CO)_4\}(CH_2Cl_2)_2][\mu\text{-}(CN)\{B(C_6F_5)_3\}_2]$ (90%) and $[Ag(CH_2Cl_2)_3][\mu\text{-}(CN)\{B(C_6F_5)_3\}_2]$ (10%).

Identification code	2471679
Empirical formula	$C_{43.7}H_{6.2}AgB_2Cl_{6.2}F_{30}NNi_{0.9}O_{3.6}$
Formula weight	1574.82
Temperature/K	100.0
Crystal system	monoclinic
Space group	$P2_1/c$
a/Å	13.2880(5)
b/Å	28.0807(10)
c/Å	13.7219(4)
α/°	90
β/°	91.6140(10)
γ/°	90
Volume/Å^3	5118.1(3)
Z	4
ρ_{calc}g/cm^3	2.044
μ/mm^{-1}	1.194
F(000)	3047.0
Crystal size/mm^3	0.1 × 0.1 × 0.1
Radiation	MoKα (λ = 0.71073)
2Θ range for data collection/°	4.152 to 50.744
Index ranges	$-15 \leq h \leq 15, -33 \leq k \leq 33, -16 \leq l \leq 16$
Reflections collected	64186
Independent reflections	9383 [R_{int} = 0.0445, R_{sigma} = 0.0279]
Data/restraints/parameters	9383/2/835
Goodness-of-fit on F^2	1.059
Final R indexes [I>=2σ (I)]	R_1 = 0.0444, wR_2 = 0.1064
Final R indexes [all data]	R_1 = 0.0595, wR_2 = 0.1188
Largest diff. peak/hole / e Å^{-3}	1.18/-0.99

References

1. Origin(Pro), *Version 2016*, OriginLab Corporation: Northhampton, Massachusetts, USA, 2016.
2. M. R. Willcott, *J. Am. Chem. Soc.*, 2009, **131**, 13180–13180.
3. L. J. B. O. V. Dolomanov, R. J. Gildea, J. A. K. Howard, H. Puschmann, *J. Appl. Cryts.*, 2009, **42**, 339–341.
4. G. Sheldrick, *Acta Cryst. A*, 2015, **71**, 3–8.
5. G. Sheldrick, *Acta Cryst. A*, 2008, **64**, 112-122.
6. K. Brandenburg, *Diamond (3.2 ed)*, Crystal Impact GbR, Bonn, 2014.
7. Persistence of Vision Pty. Ltd. Persistence of Vision Raytracer. Ltd., Persistence of Vision Pty. 2004. Retrieved from http://www.povray.org/download/.
8. S. J. Lancaster, D. A. Walker, M. Thornton-Pett and M. Bochmann, *Chem. Commun.*, 1999, 1533–1534.
9. M. K. Assefa, J. L. Devera, A. D. Brathwaite, J. D. Mosley and M. A. Duncan, *Chem. Phys. Lett.* 2015, **640**, 175–179.
10. M. J. Frisch, G. W. Trucks, H. B. Schlegel, G. E. Scuseria, M. A. Robb, J. R. Cheeseman, G. Scalmani, V. Barone, G. A. Petersson, H. Nakatsuji, X. Li, M. Caricato, A. V. Marenich, J. Bloino, B. G. Janesko, R. Gomperts, B. Mennucci, H. P. Hratchian, J. V. Ortiz, A. F. Izmaylov, J. L. Sonnenberg, Williams, F. Ding, F. Lipparini, F. Egidi, J. Goings, B. Peng, A. Petrone, T. Henderson, D. Ranasinghe, V. G. Zakrzewski, J. Gao, N. Rega, G. Zheng, W. Liang, M. Hada, M. Ehara, K. Toyota, R. Fukuda, J. Hasegawa, M. Ishida, T. Nakajima, Y. Honda, O. Kitao, H. Nakai, T. Vreven, K. Throssell, J. A. Montgomery Jr., J. E. Peralta, F. Ogliaro, M. J. Bearpark, J. J. Heyd, E. N. Brothers, K. N. Kudin, V. N. Staroverov, T. A. Keith, R. Kobayashi, J. Normand, K. Raghavachari, A. P. Rendell, J. C. Burant, S. S. Iyengar, J. Tomasi, M. Cossi, J. M. Millam, M. Klene, C. Adamo, R. Cammi, J. W. Ochterski, R. L. Martin, K. Morokuma, O. Farkas, J. B. Foresman and D. J. Fox, Gaussian, Inc., Wallingford, CT, 2016.
11. A. D. Becke, *J. Chem. Phys.* 1993, **98**, 5648-5652.
12. S. Grimme, S. Ehrlich, L. Goerigk, *J. Comput. Chem.* 2011, **32**, 1456-1465.
13. F. Weigend, A. Ahlrichs, *Phys. Chem. Chem. Phys.* 2005, **7**, 3297-3305.
14. A. D. Becke, *Phys. Rev. A*, 1988, **38**, 3098–3100.
15. J. P. Perdew, *Phys. Rev. B*, 1986, **33**, 8822–8824.
16. J. P. Perdew, *Phys. Rev. B*, 1986, **34**, 7406–7406.
17. E. Caldeweyher, S. Ehlert, A. Hansen, H. Neugebauer, S. Spicher, C. Bannwarth and S. Grimme, *J. Chem. Phys.*, 2019, **150**, 154122.
18. G. te Velde, F. M. Bickelhaupt, E. J. Baerends, C. Fonseca Guerra, S. J. A. van Gisbergen, J. G. Snijders and T. Ziegler, *J Comput. Chem.*, 2001, **22**, 931967.
19. AMS 2023.1, SCM, Theoretical Chemistry, Vrije Universiteit, Amsterdam, The Netherlands, http://www.scm.com
